# Impact of connected health on the psychological wellbeing and quality of life of people with multiple sclerosis and their caregivers: A systematic review

**DOI:** 10.1177/20552076251326230

**Published:** 2025-03-31

**Authors:** Joan Alaboson, Laura Coffey, Sowmya Shrivastava, Adeola Ade-Alao, Rebecca Maguire

**Affiliations:** 1 Department of Psychology, 8798 Maynooth University, Kildare, Ireland; 2 107738 Department of Biology, Federal University of Technology, Akure, Ondo State, Nigeria

**Keywords:** Connected health, multiple sclerosis, quality of life, psychological wellbeing, informal caregivers

## Abstract

**Background:**

Connected health (CH) interventions may improve psychological wellbeing and quality of life (QoL) in caregivers and people with multiple sclerosis (MS); however, this impact has not been rigorously evaluated. This systematic review aims to synthesize the literature assessing CH technology's impact on psychological wellbeing and/or QoL of people with MS (PwMS) and/or their caregivers.

**Methods:**

This systematic review's protocol is registered with International Prospective Register of Systematic Reviews (PROSPERO) with identification number CRD42023402434. CINAHL, Web of Science, PubMed, Embase, and PsycINFO databases were searched with terms relating to (a) CH; (b) MS; (c) psychological wellbeing/QoL; and (d) caregivers/people with MS. Of 2821 screened articles, 47 met the eligibility criteria, with just three including MS caregivers.

**Results:**

Heterogenous interventions supporting self-management (*n* = 20 studies), education (*n* = 17 studies), psychological (*n* = 14 studies) or physical (*n* = 9 studies) rehabilitation and peer support (*n* = 5 studies) were found. CH technologies had mixed effectiveness in improving psychological and QoL outcomes, with results potentially impacted by technology type, intervention and target group. The study's findings have limited generalizability to improve access across sub-national locations, with no studies disaggregating between urban and rural residence and the majority conducted in the USA and Western Europe.

**Conclusion:**

CH technologies show promise in improving psychological wellbeing and QoL among PwMS and their caregivers. However, this necessitates further study comparing connected health and MS subtypes to improve reproducibility and effectiveness.

## Introduction

Identifying ways to support the psychological wellbeing and quality of life (QoL) in people with multiple sclerosis (PwMS) is of increasing interest to both researchers and clinicians. PwMS may experience lower QoL linked to the variability and progression of multiple sclerosis (MS) symptoms, which may lead to emotional or mental challenges.^1,2^ Symptoms can include difficulties with cognition, mobility and fatigue, among others, and may be followed by periods of reprieve (relapse and remit), or progressively worsen.^3^ Variations in MS or sub-types can result in physical dysfunction and psychological distress,^4^ which may impact QoL.^5^ Compared to the wider population, PwMS have a greater risk of anxiety and depression,^6^ with a reported prevalence of 22.1% and 30.5%, respectively.^7^

Beyond the impact of MS on patients themselves, family members, who play varying roles supporting PwMS as informal caregivers,^8^ may experience lower psychological wellbeing as a result of their caring role.^9,10^ In Europe, 46% of PwMS receive informal care,^11^ for an average of three hours daily, with a potentially lifelong commitment.^12^ Although some research on caregiver burden illustrates both positive and negative effects of caring for PwMS,^13^ few studies have explored ways to better support caregivers’ psychological wellbeing and/or QoL.

To address these problems in the context of MS, psychological approaches such as mindfulness^14^ or cognitive behavioural therapy (CBT)^15^ may help. However, PwMS may be prevented from availing these services.^16^ These barriers could be systemic, such as the shelter-in-place COVID-19 measures,^17^ which impacted on the psychological wellbeing of PwMS and caregivers.^18^ PwMS could also experience reduced mobility and/or disabilities that result in further needs for mobility or transportation accommodations^4,19^ and service flexibility,^20^ that can further hinder access to needed services.

Connected health (CH), an umbrella term involving internet-mediated technologies that enable two-way communication, information processing and analysis, often between two parties,^21^ may play a role in improving access to healthcare.^22^ CH includes a wide range of technologies such as websites, mobile applications, wearables, social media and messaging applications, among others. Further, numerous other terminologies can be categorized as CH, including digital health, electronic health and wearable devices.^23^ Taken together, research in this area has presented a fragmented view of how these technologies have been used among PwMS.

In previous reviews, Scholz and colleagues focused on eHealth excluding mobile applications, social media and gamified technology.^24^ They assessed acceptance factors influencing successful use, finding user-centred design was a key determinant of the acceptance of eHealth interventions among PwMS.^24^ Hessen and colleagues focused on randomized controlled trials of self-guided mobile applications excluding websites, wearables and other technologies.^25^ They found a predominance of pilot studies, with significant results on outcomes including depression, but reported challenges with the methodological rigor of included studies.^25^ These provide evidence about the potential for some components of CH technology in this area.

Where CH interventions were assessed in the context of MS, a report focusing on both health workers and PwMS was conducted.^26^ However, to our knowledge, no systematic review has broadly examined evidence on the impact of CH interventions on PwMS. Gathering evidence in this area is particularly important for informing the provision of health and social care services, which have increasingly adopted various forms of technology since the COVID-19 pandemic.^17^

There is limited assessment of the impact of CH interventions in MS, with either a direct or indirect focus on psychological outcomes. While some technologies have facilitated self-management,^27^ symptom management and physical activity promotion,^26^ there is paucity of evidence aggregating the impact of CH on psychological outcomes. Finally, to our knowledge, no review has explored the impact of these technologies on informal MS caregivers, who we define as any person providing support (physical, emotional or social) outside healthcare professionals, at home or within the community. Our review addresses these knowledge gaps. Specifically, this systematic review aims to synthesize the literature assessing CH technology's impact on psychological wellbeing and/or QoL of PwMS and/or their informal caregivers.

## Methodology

This review employed the Preferred Reporting Items for Systematic review and Meta-Analysis (PRISMA) guidelines.^28^ Its protocol is registered with the International Prospective Register of Systematic Reviews (PROSPERO) with identification number CRD42023402434. The systematic review did not deviate from the protocol.

### Search strategy

CINAHL, Web of Science, PubMed, Embase and PsycINFO were searched for articles published between January 2013 and April 2023, with terms relating to (a) CH; (b) MS; (c) psychological wellbeing or QoL; and (d) patients or caregivers. The selected databases are repositories for medical (CINAHL and PubMed), psychology (Embase and PsycINFO) and general science (Web of Science) articles. Terms were informed by previous literature.^21,29–32^ Subject headings, MeSH, EMTREE and equivalents were searched, with findings broadened using exploded terms.

### Selection criteria

Studies reporting primary data evaluating CH technology in PwMS aged ≥18 years and/or their caregivers, with psychological wellbeing and/or QoL reported as a primary or secondary outcome using validated measures, were included. All study designs evaluating a CH intervention and its effects on psychological wellbeing and/or QoL were included. Reviews, commentaries and other descriptive designs were excluded. Only those published in peer-reviewed journals in English within the past 10 years were included, reflecting the technological advancements within the period. Grey literature were excluded to ensure that conclusions were based on studies with a higher chance of methodological rigor, transparency and reproducibility. The addition of grey literature may improve methodological quality but studies are inconclusive,^33^ and the risk of inability to access and retrieve included studies is higher since there are no general repositories for them.^34^

Articles were excluded if any of the above inclusion criteria were not met, as were articles focusing on more than one disease that did not conduct a separate analysis on PwMS and/or their caregivers. Formal paid caregivers or healthcare professionals were also excluded (see supplementary materials for detailed search strategy and inclusion/exclusion criteria).

### Screening

Database search results were exported to Endnote and then imported into Rayyan.^35^ After duplicate removal, two reviewers (JA and AA) independently screened titles and abstracts for eligibility. Full texts of potentially eligible papers were independently reviewed by two reviewers (JA and AA/SS). Conflicts over eligibility were discussed between reviewers or arbitrated by LC or RM to achieve consensus.

### Data extraction

JA systematically extracted data from included studies (all studies were cross-checked by AA). Using Microsoft Excel, the following data were collated: general description (including title, author, and publication year), study design and population type (including sample characteristics), CH type, outcome/measures used and results reported.

### Methodological quality assessment

JA and AA independently assessed the quality of included studies using the Mixed Methods Appraisal Tool (MMAT:^36^ which categorizes studies based on methodology and critically assesses quality based on design). Each study is screened by two questions and five design-specific questions (relating either to qualitative, quantitative randomized controlled trial, quantitative non-randomized, quantitative descriptive or mixed-methods studies). These generally included questions on the rationale; clarity and focus of the research question; appropriateness of the methodological approach, measures and analytical techniques used; completeness of findings and interpretation of results; and others.^36^ Included studies were assessed with criteria specific to their methodological design.

Reviewers interpreted studies as high quality with 4–5 criteria met, moderate quality with 3 criteria met, and low quality with 0–2 criteria met, in line with MMAT guidelines.^36^ Quality appraisals of each study were conducted to aid readers’ critical consideration of the credibility of their findings. No studies were excluded on this basis.

### Synthesis of findings

Articles were initially assessed to determine homogeneity and availability for meta-analysis. The observed study diversity determined thematic analysis as appropriate to further analyse findings.^37^ Data were aggregated into themes to identify connections and understand the impact of CH technologies on the psychological wellbeing and/or QoL of PwMS, and/or their caregivers. Analysis was staggered, with coding of outcomes, interventions and CH technologies done sequentially.

Initial coding frames, informed from the data, were developed by JA and reviewed by RM and LC. With consensus reached, similar codes were thematically grouped, and further consensus was ensured through discussion. Evidence patterns were captured in themes that enabled interpretation through the synthesis of findings on CH technology's impact on psychological wellbeing and/QoL. Themes were described narratively to summarize the general evidence as well as to account for CH technology's impact within each thematic area generated from analysis.

## Results

### Search outcome

**Figure 1.**
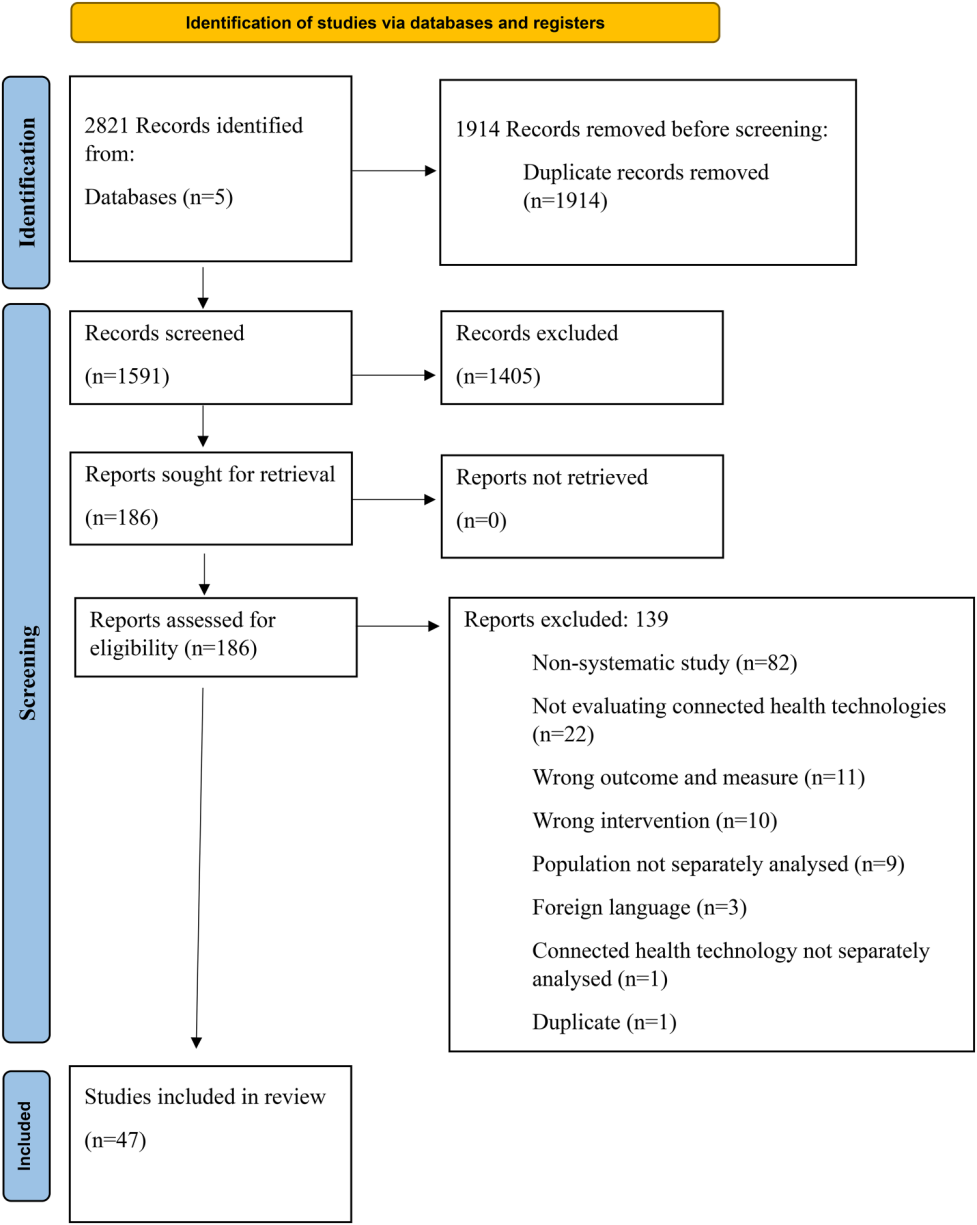
Systematic review PRISMA flow diagram.

The database searches resulted in 2821 articles retrieved, with 1591 identified for title and abstract screening following de-duplication. Of these, 186 full texts were assessed for eligibility, and 139 were excluded (see Figure 1).

### Quality appraisal findings

Using the MMAT, 34 studies achieved a high-quality assessment; eight were of moderate quality, while five were of low quality. Evaluation results reflected variability in reported methodology and analysis of included studies. Reasons for studies’ unmet quality criteria included unclear/no information on randomization, lack of assessor blinding and participant compatibility and poor reported participant adherence to the designed intervention. Of the five studies deemed to be of low quality, four were randomized controlled trials (RCT).

### Study characteristics

A description of the 47 included studies is presented in Table 1 (see supplementary materials for detailed overview). Of these, 28 were clinical trials, with three single-group trials, one two-group trial, one non-randomized control trial and 23 randomized with controls. Five studies employed a pre–post-experimental design, two used mixed methods and 11 were observational (primarily cohort) studies. One study was a natural experiment. Only six studies reported qualitative findings, largely as open-text responses to survey questions.

**Table 1.** Summary characteristics and demography of included articles.

Authors and year	Study design	Sample, including MS type (RRMS %) of intervention participants, if applicable	Gender (female %) and age (mean/range (SD))	Intervention focus	Effect on psychological wellbeing	Effect on—QoL	MMAT
Alschuler and colleagues, 2018 ^44^	RCT	PwMS (*n* = 31); 83%	83.30%; 59.8 (7.7)	Psychoeducation	None		High-4
Bessing and colleagues, 2022 ^41^	Cohort	PwMS (*n* = 213)); Nr No MS (*n* = 347);	80.75%; PwMS: 50.68 (10.98) No MS: 52.23 (12.92)	MOOC		None	Moderate-3
Boeschoten and colleagues, 2012 ^45^	Pre–post	PwMS (*n* = 44); 48%	77%; 45 (12)	Psychoeducation/self-management	Positive		High-4
Bogosian and colleagues, 2015 ^46^	RCT	PwMS (*n* = 40); [42%*] [57.5%**]	47.40%; 53.42 (8.3)	Cognitive therapy	Mixed ^O^		High-5
Bulbul and colleagues, 2023 ^47^	RCT	PwMS (*n* = 50); Nr	100%; 36.76 (9.32)	Telerehabilitation/feedback		Positive	High-4
Cavalera and colleagues, 2019 ^48^	RCT	PwMS (*n* = 139); 94% [6%**]	67%; 42.26 (8.35)	Psychoeducation	Positive ^OO^	Positive ^OO^	High-5
Chen and colleagues, 2023 ^49^	Cohort	PwMS (*n* = 45); 86.67% [6.67%*] [4.44%**] [2.22%***]	91.11%; 41.69 (13.39)	Monitoring	Mixed		High-5
Chikersal and colleagues, 2022 ^50^	Experimental	PwMS (*n* = 56); Nr	86%; 43.5 (37–52)	Monitoring	Positive		High-5
Claflin and colleagues, 2023^51^	Cohort	PwMS (*n* = 813); Nr	80.60%; 47.0 (11.9)	MOOC		Positive	Moderate-3
Claflin and colleagues, 2022 ^39^	Cohort	Both^ PwMS (*n* = 213)^X^; Nr Caregiver (*n* = 144))	85.50%; 51.6 (12.2)	MOOC		None	Moderate-3
Dogru-Huzmeli and colleagues, 2021 ^52^	Observational	PwMS (*n* = 1); 100%	0; 39	Telerehabilitation		Positive	High-4
Donkers and colleagues, 2020 ^53^	RCT	PwMS (*n* = 48); Nr	63%; 54.6 (11.9)	Telephysiotherapy	Positive		High-5
Dunne and colleagues, 2021 ^54^	MM	PwMS (*n* = 55); Nr	82.40%; M4MS: 44.6 (10.1) Yoga: 48.2 (10.4)	Mindfulness	None	None	High-4
Fischer and colleagues, 2015 ^55^	RCT	PwMS (*n* = 90); 47% [16%*] [20%**] [11%***] [7%**^+^**]	76%; 45·36 (12·64)	Tele-CBT/education	Positive	None	High-4
Flachenecker and colleagues, 2020 ^56^	RCT	PwMS (*n* = 84); 56%	65%; 47.6 (9.2)	Tele-exercise/diary		Positive^!^	High-4
Gandy and colleagues, 2022 ^57^	RCT	PwMS (*n* = 85); Nr	Nr; > 18 ^XX^	Tele-CBT/education/self-management	Positive		Moderate-3
Gandy and colleagues, 2020 ^58^	CT	PwMS (*n* = 29); Nr	Nr; > 18 ^XX^	Tele-CBT/education/cognitive rehabilitation	Positive^!^		High-4
Golan and colleagues, 2021 ^59^	Cohort	PwMS (*n* = 97); 93% [1%*] [6%**]	66%; 40.4 (11)	Diary	Negative	Positive	High-4
Halstead and colleagues, 2020 ^40^	Cohort	Both^ PwMS (*n* = 31); *N* = 29 [*N* = 1*] [*N* = 1**] Caregivers (*n* = 31)	80.60%; 18–65	Psychoeducation	Positive^!!^		High-5
Jeong and colleagues, 2021 ^60^	RCT	PwMS (*n* = 45); Nr	79.30%; 57.8 (11.9)	Telerehabilitation/self-management		Positive^!^	High-4
Jongen and colleagues, 2015 ^61^	Pre-post	PwMS (*n*-105); Nr	82.80%; 44.2 (10.6)	Self-management		Positive	High-4
Kahraman and colleagues, 2020 ^42^	RCT	PwMS (*n* = 50); Nr No MS (*n* = 20)	80%; 34.5	Telerehabilitation	Positive	Positive	High-5
Kever and colleagues, 2022 ^62^	Non-randomized CT	PwMS (*n* = 31); Nr	90%; 39.5 (median)	Support	Mixed	None	High-4
Khazaelli and colleagues, 2019 ^38^	Pre-post	Caregivers (*n* = 30); Not applicable	100%; 20–70	Cognitive therapy/support	Mixed		Low-1
Kratz and colleagues, 2021 ^63^	CT	PwMS (*n* = 20); 50% [15%*] [20%**] [15%***]	65%; 48.05 (12.16)	Symptom monitoring/tele-CBT	None		High-5
Landtblom and colleagues, 2019 ^64^	RCT	PwMS (*n* = 93); 100%	63%; 41 (13.2)	Information/diary		None	Moderate-3
Leavitt and colleagues, 2020 ^65^	RCT	PwMS (*n* = 24); Nr	64.30%; 43.0 (10.8)	Support	Positive		Low-2
Limmroth and colleagues, 2022 ^66^	Cohort	PwMS (*n* = 62); Nr	66%; 43.2 (11.5)	Cognitive games		None	High-4
McArthur and colleagues, 2023 ^67^	Pre-post	PwMS (*n* = 18); Nr	89%; 64.5 (10.71)	Fall prevention course/support		None	High-4
Moss-Morris and colleagues, 2012 ^68^	RCT	PwMS (*n* = 25); 43.5% [8.7%*] [30.4%**] [17.4%***]	69.60%; 40.14 (17.76)	Educational/tele-CBT/self-management	Positive		Low-1
Najafi and colleagues, 2023 ^69^	RCT	PwMS (*n* = 45); 100%	100%; Tele-pilates: 36.2 (4.33) Tele-yoga: 37.4 (6.03)	Tele-yoga/pilates	Positive	Positive	Moderate-3
Pagliari and colleagues, 2021 ^70^	RCT	PwMS (*n* = 70); Nr	60%; 48.33 (9.66)	VR cognitive training	Positive^!!!^	Positive	High-5
Paul and colleagues, 2014 ^71^	RCT	PwMS (*n* = 30); *N* = 11 [*N* = 1*] [*N* = 2**] [*N* = 1***]	80%; 50.8 (7.4)	Self-management	None	None	High-4
Pottgen and colleagues, 2022 ^72^	RCT	PwMS (*n* = 288) ; **Intervention group 1 (IG1):** 71% [15%*] [14%**] **IG2:** 87% [3%*] [10%**]	69%; **IG1**: 42.77 (10.23) **IG2**: 43.24 (9.29)	Education/neuropsychological	Positive ^OO^	Positive ^OO^	Low-2
Pratap and colleagues, 2020 ^43^	Observational	PwMS (*n* = 526); **Self-reported** 83.6% [9.5%*] [7%**] **Referred** 90.4% [4.4%*] [3.7%**] [1.5%***] No MS (*n* = 134)	73.30%; **Self-reported** 45.20 (11.64) **Referred** 48.93 (11.20)	Self-functional assessments		None	High-5
Sadeghi and colleagues, 2022 ^73^	RCT	PwMS (*n* = 60); *N* = 21 [*N* = 1*] [*N* = 8**]	63.30%; 41.3 (10.4)	Telemedicine	None	None	High-4
Saladino and colleagues, 2023 ^74^	Cohort	PwMS (*n* = 68); **IVR**: *N* = 42 [*N* = 4*] [*N* = 8**] **TR:** *N* = 13 *N* = 1*	57.40%; **IVR**: 44.7 (18–77) **TR**: 41.7 (25–58)	Neurorehabilitation/telerehabilitation	Positive	Positive	High-5
Sangelaji and colleagues, 2017 ^75^	MM	PwMS (*n* = 4); *N* = 1 [*N* = 2**] [*N* = 1***]	100%; 76; 65; 56; 56	Telephysiotherapy/diary		Positive	Low-1
Sesel and colleagues, 2022 ^76^	RCT	PwMS (*n* = 132); 86.96% [4.35%*] [2.9%**] [5.8%***]	Nr; 45.13 (10.74)	Mindfulness	Mixed	Positive	Moderate-3
Tarakci and colleagues, 2021 ^77^	RCT	PwMS (*n* = 41); 100%	73%; 39.46 (10.59)	Telerehabilitation		None	High 4
Turkowitch and colleagues, 2022 ^78^	CT	PwMS (*n* = 21); 100%	81.80%; 50.3 (13.5)	Tele-CBT	None	None	High-4
Turner and colleagues, 2016 ^79^	RCT	PwMS (*n* = 64); 65.5%	29%; 52.7 (11.6)	Telehealth/tele-counselling	Positive		High-5
Van Beek and colleagues, 2022 ^80^	RCT	PwMS (*n* = 48); 65% [23%*] [12%**]	81%; 50.84 (14.84)	Self-management/VR tele-exercises		Positive ^O^	High-5
Van Geel and colleagues, 2020 ^81^	Pre-post	PwMS (*n* = 12); *N* = 11 [*N* = 1**]	100%; 42.5	Self-management/support		None	High-4
Van Kessel and colleagues, 2016 ^82^	RCT	PwMS (*n* = 39); 79.0% [10.5%**] [10.5%***]	58%; 42.95 (8.16)	Information/self-management	None		Moderate-3
Wingo and colleagues, 2020 ^83^	Observational	PwMS (*n* = 20); 100%	85%; 46.15 (11.60)	Education/tele-coaching/diary	Mixed		High-4
Zissman and colleagues, 2012 ^84^	CT	PwMS (*n* = 40); 100%	85%; 43.8 (11.5)	Information		Positive^!!!^	High-4

CBT: cognitive behavioural therapy; CT: Clinical trial; diary: for tracking progress or symptom monitoring; HRQoL: health-related quality of life; IG1: intervention group 1; IG2: intervention group 2; IVR: institutional virtual reality; M4MS: mindfulness for multiple sclerosis; MM: mixed methods; MOOC: massive open online course; MSP: MySupportPlus; *N* or *n*: numbers; Nr: not reported; QoL: quality of life; RCT: randomized controlled trial; SD: standard deviation; TR: telerehabilitation; VR: virtual reality; ^O^: positive results not sustained at 3-month follow-up; ^OO^: positive results not sustained at 6-month follow-up; ^X^: demography provided for completers; ^XX^: no separate analysis of demography done; *: PPMS; **: SPMS; ***: unsure/unknown MS type; ^: both caregivers and PwMS; !: results sustained at 3 and/or 6-month follow-up; !!: both caregivers and PwMS; !!!: both intervention and control-reported positive changes.

Three quarters of included articles (*n* = 35) were published since 2019, suggesting increased study on this topic in the past 5 years. Studies were conducted mainly in North America and Europe, with 13 studies based in the USA. Australia was the second most frequent study location, with six papers identified; however, a total of 15 countries were included.

Studies included a total of 4871 participants (ranging from *n* = 1 to 813), of whom the majority (*n* = 4165) were PwMS. Three studies included caregivers (total caregiver *n* = 205). Of these, only one^38^ focused on caregivers alone while^39,40^ including both PwMS and caregivers. A further three studies^41–43^ compared PwMS to a population without MS (*n* = 501), while the remaining 41 studies had PwMS as a single population.

Participants were predominantly female and, for studies reporting mean age, ranged from 35 to 65 years old. The average time living with MS ranged from 5 to 41 years. Most (*n* = 29) studies reported participants’ MS type, with 15 including participants of all phenotypes, relapsing–remitting MS (RRMS), primary progressive MS (PPMS), secondary progressive MS (SPMS) and unknown MS type. Of the remaining 14 studies, eight included only RRMS participants, and a further five included participants reporting either RRMS or SPMS. Just one study only focused on people with progressive MS. Thirty studies reported participants’ disability status, 21 using the Expanded Disability Status Scale (EDSS), with a mean EDSS range of 2.5–6.8. Other studies (*n* = 9) employed Patient Determined Disease Steps (PDDS) and ambulation status to quantify mobility levels. Full details were provided in the supplementary material.

## Measures

Overall, 16 studies included a measure of psychological wellbeing, 16 a measure of QoL and 15 a measure of both. Twenty-seven studies evaluated anxiety and/or depression, with the most frequently used measure being the Hospital Anxiety and Depression Scale (HADS) in 13, the Patient Health Questionnaire (PHQ-9) in nine, and the Beck Depression Inventory (BDI) in seven studies.

Assessment of QoL or health-related quality of life (HRQoL) was made using the MS Impact Scale (MSIS-29) in 11 studies and the MSQoL-54 in seven. Further details are included in supplementary materials.

## Description and nature of interventions

Table 1 summarizes the interventions’ focus. Overall, 13 studies had a physical exercise focus, including six on general exercise, two on physiotherapy and one each on yoga and pilates, fall prevention, walking, Cawthorne–Cooksey rehabilitation and pelvic floor muscle training. Other study interventions delivered, singly or in combination, psychologically based approaches (*n* = 14), MS symptom management or other telehealth services (*n* = 20) or were designed as a source of information (*n* = 17).

Intervention durations ranged from 3 to 312 weeks, with an average duration of 20 weeks. However, most (*n* = 11) lasted 12 weeks. Of the 29 studies with controls, five had a waitlist, five had usual/standard care and three had an active comparator, while technical support, education and no treatment were controls in one study each. Participant engagement in study interventions varied, ranging from 10- to 90-minute sessions, once to several times weekly.

## Connected health technologies

A range of CH technologies were employed in studies. In most cases, one mode of CH technology was deployed; however, seven^38,48,49,57,64,68,69^ studies deployed an intervention with two CH technologies, and one^83^ study deployed three CH technologies. Websites or web-based programs were the most frequently used single CH technology (*n* = 17). These were used to deliver support groups in two^62,65^ studies, self-management in five^45,61,63,71,82^ studies, education in six^39–41,51,55,58^ studies and various other services (e.g. exercise activity monitoring) in four^53,75,76,79^ studies. A wide combination of outcomes were targeted here, mainly combinations of anxiety, depression and/or QoL.

CH-mediated teleconference (mediated entirely by the internet) was the second most frequently employed (*n* = 12), mainly to deliver services (primarily rehabilitation but also provision of psychological support) in ten^42,46,47,52,54,60,69,73,77,78^ studies. Of these, half ^42,47,52,60,77^ delivered telerehabilitation, with two other studies supporting learning or education.^67,84^

Four additional studies deployed teleconference in combination with other CH technologies. These delivered self-management,^49^ education,^48^ psychological support (mindfulness)^38^ and tele-yoga or pilates.^69^ Teleconferencing-mediated interventions mainly focused on improving QoL, as well as anxiety and depression.

Other CH technologies used included mobile apps (*n* = 5), tablet app (*n* = 1), VR (*n* = 2) and online computer software (*n* = 2). Where multiple CH technologies were employed, teleconference was a frequent component (see supplementary materials for further details).

## Narrative synthesis

Psychological patient-reported outcomes included anxiety, depression, wellbeing and positive affect, as well as QoL and HRQoL. Several studies targeted these outcomes using a combination of CH technologies (see supplementary materials). The impact of interventions on these outcomes was categorized based on five thematic clusters: (a) self-management; (b) education or information provision; (c) psychological-based interventions; (d) telemedicine and telerehabilitation; and (e) support groups.

## Self-management, assessment, diary feedback and symptom monitoring

Twenty studies assessed the impact of self-management/assessment, symptom monitoring and feedback on psychological wellbeing and/or QoL. Many studies delivering self-management and digital diaries utilized online or web-based programs (*n* = 8),^45,56,61,63,71,75,79,82^ a combination of website and email (*n* = 3)^57,64,68^ or website and study app (*n* = 1).^83^ Mobile apps were the second most frequently used CH technology to promote self-management interventions (*n* = 4).^43,50,59,81^

These interventions involved an interface where self-management was promoted and included providing educational materials,^45,64,68^ virtual coaching or consultation^56,61,81–83^ or psychological support.^57,63^ Three studies reported that interventions were supported by telephone calls^64,68,79^ or reminders that were sent to participants via text message.^59,64,81^

Overall, the reported impact of self-management interventions on psychological wellbeing and/or QoL/HRQoL was mixed, with some positive effects observed on anxiety, depression and QoL. CH technologies utilizing self-management saw improvements in anxiety in three of five studies, depression in four of six studies and QoL or HRQoL in three of five studies. Digital diary use was associated with improvement in QoL/HRQoL in five of seven studies, although no change in anxiety or depression was observed (see Table 1 and supplementary materials for details).

One study using Fitbit sensors with a mobile app in symptom monitoring noted improvement in depression detection.^50^ Another study utilizing sensors on a smartphone and mobile app found no change in QoL between participants and controls.^43^ A 12-week RCT evaluating the effectiveness of a web-based personalized program along with a diary on anxiety, depression and QoL noted no improvement compared to usual care.^71^ There was, however, an unexpected reduction in anxiety among controls.^71^ No studies providing self-management interventions included caregivers.

In open-text responses, participants using a website for self-management revealed that connecting remotely was easy or convenient to engage with.^71^ Some also discussed disadvantages they found and offered practical suggestions for improvements.^71,75^

## Education or information provision

Seventeen studies assessed the impact of CH-delivered information and/or education on QoL, HRQoL, anxiety, depression, emotional wellbeing and/or mood, with mixed reported efficacies. Websites were the most frequently used CH technology in the delivery of these interventions.^39–41,45,48,51,55,57,58,64,68,82,84^ However, other media, namely, emails,^67,83^ software,^72^ teleconferences^44^ and a call centre,^84^ were also used to disseminate information and education. Sixteen studies involved delivery of courses or online programs.^39–41,44,45,48,51,55,57,58,64,67,68,72,82,83^ Of these, one was a physical or occupational therapist-led course on fall prevention,^67^ and the other deployed psychologists delivering positive psychology content.^44^ Of 14 studies involving self-directed courses, four employed additional support through telephone calls^68^ or website communication^45^ with psychologists, remote clinicians^57^ or a tele-coach.^83^

Providing information or education using CH technologies was shown to have mixed but generally promising positive effects on both anxiety and depression and had a more positive effect on QoL than HRQoL. Overall, 4 of 7 studies reported improvements in QoL,^48,51,55,72^ 1 of 3 in HRQoL^84^ and 7 of 10 in anxiety^40,45,48,57,58,68,72^ and depression.^45,48,55,57,58,68,72^ Among these, improvements were found in participants with concurrent diagnosis of major depressive and/or anxiety disorder following a 5-week course focused on problem-solving.^45^ However, an RCT evaluating the effectiveness of MSexpertise, a psychologist-led program on positive psychology with waitlist control, did not affect anxiety, depression or QoL.^44^ Conversely, QoL improved following a longitudinal cohort study using a massive open online course (MOOC) over 6 weeks.^51^ Despite other studies reporting improvements in anxiety, depression and QoL following CH technology-mediated education or information provision, effects were not sustained at 6-month follow-up in two studies, including an 8-week group psychoeducation course^48^ and a 3-week combined MS education, exercise and psychological treatment program.^72^

CH-mediated education or information provision had low efficacy in caregivers, with studies failing to report changes in psychological wellbeing or QoL.^39,41^ However, a cohort study with participating caregiver–PwMS dyads that delivered web-based education on practical skills and opportunities through role play found improvements in anxiety, emotions and life satisfaction in both groups.^40^ Further, through open text, participants shared both self-empowering and relationship-strengthening experiences following their participation in the program.^40^

## Psychological support, training and rehabilitation

Fourteen studies employed mindfulness, cognitive training, CBT or other psychological support either singly or with other therapies using CH technologies (see Table 2). Five of these utilized websites or online programs,^55,57,58,68,76^ five used teleconferencing,^38,44,46,54,78^ three utilized software^72^ including VR^70,74^ and one used mobile app games.^66^ In some studies, participants connected with psychology professionals directly (e.g. using teleconferencing) or indirectly (using software). In others, simulations through games or VR were used.

**Table 2.** Psychological intervention focus, CH technology and outcomes.

Author and year		Psychological intervention	CH technology	Control intervention	Duration (weeks)	Outcome			Follow-up
						Positive effect	No effect	Negative effect	
One intervention supported by CH technology	Moss-Morris and colleagues, 2012 ^68^	CBT	Website	Standard care	10	Anxiety Depression			Lower at 10 weeks
	Turkowitch and colleagues, 2022 ^78^	CBT	Teleconferencing	Face-to-face CBT	6		QoL Depression Anxiety		
	Gandy and colleagues, 2022 ^57^	CBT	Website and email	No treatment	10	Anxiety Depression			
	Fischer and colleagues, 2015 ^55^	CBT	Website	No treatment	9	QoL Depression			
	Alschuler and colleagues, 2018 ^44^	Positive psychology skill training	Teleconferencing	Waitlist			Depression Anxiety Positive affect wellbeing		
	Limroth and colleagues, 2022 ^66^	Cognitive training/neurorehabilitation	Mobile app		52		HRQoL		None at 3 and 6 months
	Pagliari and colleagues, 2021 ^70^	Cognitive training/neurorehabilitation	Virtual reality	Usual care	6	QoL	Depression		
	Saladino and colleagues, 2023 ^74^	Cognitive training/neurorehabilitation	Virtual reality software and teleconferencing	VR software	24	Depression QoL			
	Sesel and colleagues, 2022 ^76^	Mindfulness	Website	Waitlist	8	Depression HRQoL	Anxiety		
	Dunne and colleagues, 2021 ^54^	Mindfulness	Teleconferencing	Waitlist	8		QoL Wellbeing		
Two interventions supported by CH technology	Pottgen and colleagues, 2022 ^72^	Cognitive training/neurorehabilitation. Psychological treatment	Software	Usual care	3	Anxiety Depression QoL			None at 6 months
	Gandy and colleagues, 2020 ^58^	Cognitive training/neurorehabilitation. CBT	Website		6	Anxiety Depression			Lower at 3 months
	Bogosian and colleagues, 2015 ^46^	Mindfulness Cognitive training/neurorehabilitation	Teleconferencing	Waitlist	8	Psychological distress Anxiety	Depression		Lower psychological distress, but not anxiety, at 3 months
	Khazaeili and colleagues, 2019 ^38^	Mindfulness Cognitive training/neurorehabilitation	Teleconferencing	No intervention	8	Anxiety	Depression		

### Cognitive training and neurorehabilitation

Half the studies in this thematic cluster (*n* = 7) deployed cognitive training or neurorehabilitation, either singly or in combination with other psychological support. In single deployment, cognitive training and neurorehabilitation had mixed effects, generally improving QoL but not HRQoL, and in one of two studies, lowering depression. A cohort study observing the effect of telerehabilitation along with VR exergame software for cognitive training reported lower anxiety and depression symptoms after 24 weeks, compared to VR software alone.^74^ However, in another 6-week study employing VR, QoL but not depressive symptoms improved compared to usual care.^70^ There was also no change in HRQoL following a mobile app, gamified, cognitive training intervention after 52 weeks or at follow-up 3 and 6 months later.^66^

CH-mediated interventions deploying two types of psychological support including cognitive training and/or neurorehabilitation showed promise for improving QoL, anxiety and psychological distress, but not for depression. Anxiety and depression symptoms were reduced in two studies, including a software-mediated psychoeducation program with neuropsychological exercises and treatment,^72^ and a website deploying CBT and neurorehabilitation in a single-group open trial.^58^ Both studies recorded sustained impacts at 6- and 3-month follow-up. In contrast, an RCT evaluating effectiveness of teleconferencing-enabled cognitive training and mindfulness resulted in reduced anxiety and psychological distress but not depression, compared to a waitlist control.^46^ Similarly, a quasi-experimental study evaluating teleconference-mediated mindfulness and cognitive therapy on caregivers alone reduced anxiety but not depression.^38^

### Cognitive behavioural therapy or other psychological support

Six studies employed interventions involving CH-mediated CBT or positive psychology. Most involved the study of a single psychological intervention or CBT modality, while one was combined with cognitive training. Interventions involving CH-mediated CBT alone using websites or software led to positive effects on anxiety, depression and QoL. These included a 10-week self-managed CBT program, which demonstrated improvements in anxiety and depression compared with standard care, sustained at 10-week follow-up,^68^ and a 10-week website-mediated CBT intervention, supported by email or telephone support, which resulted in lower anxiety and depression compared to no treatment.^57^ However, one study found teleconference-enabled CBT had no effect on QoL, depression and anxiety compared to face-to-face delivery after 6 weeks.^78^

Only one study utilized website-mediated CBT along with cognitive training, with positive effects on anxiety and depression, sustained at 3 months.^58^ There was no effect on anxiety, depression, positive affect and wellbeing where teleconferencing-mediated positive psychology skills training was compared to waitlist intervention.^44^

### Mindfulness

Four studies examined the efficacy of CH-mediated mindfulness intervention either alone (*n* = 2) or in combination with cognitive therapy (*n* = 2). Teleconferencing was employed in three studies evaluating mindfulness, with mixed effects. Mindfulness delivered as a single CH-mediated psychological intervention had low efficacy in improving psychological outcomes.^54,76^

Where delivered in combination with another psychological intervention, CH-mediated mindfulness therapy was efficacious in reducing anxiety but not depression.^46^ Similarly, anxiety but not depression improved following a teleconference-mediated mindfulness and cognitive therapy intervention with caregivers.^38^

## Telemedicine and telerehabilitation

Nine studies explored the impact of CH technology-supported medical, exercise or other physical activity-related telerehabilitation services on psychological wellbeing and QoL. Teleconferencing was solely employed in six of these studies,^42,47,52,69,77^ including one involving teleconsultation.^73^ Participants received exercise instructions or physical activity through teleconferencing, websites or software or consultations from therapists live or through video recordings.

The impact of CH-mediated rehabilitation or care provision on psychological outcomes was mixed, but improvements in QoL were observed most frequently. An RCT evaluating web software to plan and record physical activity improved HRQoL after 12 weeks compared to usual care, sustained at 3- and 6-month follow-up.^56^ Further, a trial of pelvic floor muscle training improved QoL following completion compared to controls,^47^ while a case study reported QoL improvements of a participant receiving diplopia rehabilitation exercises (Cawthorne–Cooksey) through teleconference.^52^

Conversely, a trial providing teleconsultation through teleconferencing in addition to standard care did not improve HRQoL, anxiety or depression compared to controls over 52 weeks.^73^ Another RCT exploring the efficacy of teleconference-delivered structured exercise compared to controls receiving rehabilitation with a physical therapist found no difference in QoL after 12 weeks.^77^

## Support groups

Five studies evaluated the impact of CH-mediated support groups on psychological outcomes including mood,^65^ QoL,^62,67^ HRQoL,^81^ anxiety,^38,62^ and depression.^38,62^ CH modalities included teleconference or online meetings^62,65,67^ (*n* = 3), anonymous interactions on study app^81^ (*n* = 1) and social media using Telegram software^38^ (*n* = 1). The efficacy of CH-mediated support group interventions varied across studies and outcomes, with some effect in alleviating either anxiety or depression symptoms but not QoL.

In one study involving only caregivers of people with RRMS, social media was used for 8 weeks to provide educational materials complementing teleconference mindfulness-based therapies, resulting in lower anxiety but not depression in the intervention group.^38^ In another study, an app, WalkWithMe, designed to promote physical activity through walking and alleviate fatigue and cognitive symptoms used anonymous support to enhance participation and motivation over 10 weeks and was not associated with a change in HRQoL.^81^

In two of the three studies using teleconferencing, participants were encouraged to share positive and negative aspects of living with MS and in one^62^ discussions were also led by a psychologist. While no change in QoL was observed in either of these studies,^62,67^ Kever and colleagues (2022) reported lower anxiety but not depression. Another study using teleconferencing with a 12-week-long private online group did not impact QoL.^65^

Generally in open-text responses, participants shared positive feelings of connectedness to peers through support groups.^40,44,65,75^ Support groups provided a safe and intimate space, as well as an opportunity to learn from others.^65^

## Discussion

This is the first systematic review to examine the impact of CH technologies on the psychological wellbeing and QoL of PwMS and their caregivers. A wide variety of CH technologies were employed across the 47 studies included, but these were mostly limited to websites and teleconferencing either alone or in conjunction with other technologies. Overall, findings suggest that CH technologies could be successful in improving psychological wellbeing and/or QoL in PwMS. However, there is a need for further robust evaluation, given the diversity of CH technologies, interventions, measures and outcomes utilized across studies. Further, only three studies included caregivers as participants, revealing a paucity of evidence supporting the effectiveness of CH technology-driven interventions in promoting psychological wellbeing in this population.

We found that interventions delivered using CH technologies have potential to support the psychological wellbeing and/or QoL of PwMS, but this was nuanced by several factors. First, the type of CH technology employed appeared to influence the impact of interventions. For instance, CH-mediated mindfulness-based interventions delivered using web programs were more effective in improving depression in PwMS than teleconferencing. Second, CH technology was more effective in delivering some types of interventions than others. For example, teleconference-mediated telemedicine and telerehabilitation focused on physical exercise or rehabilitation frequently improved QoL, while CH-mediated support groups reported no such effect. Similarly, CH-mediated interventions may have a comparable effect to traditional (in-person) approaches. For instance, CH-mediated psychological support had positive effects on psychological outcomes compared to standard care. Finally, the effects of CH-mediated interventions may depend on whether the end user is a PwMS or caregiver. For example, we found that CH-mediated education or information provision had lower efficacy in improving caregiver QoL, anxiety or depression, whereas this was often associated with improvements in anxiety, depression and QoL/HRQoL in PwMS.

Our review builds on existing knowledge about CH technology use and impact in MS. We found that QoL often improved in PwMS following telemedicine or telerehabilitation focused on exercise and physical activity. In another review, higher engagement in physical activity was found to be increase the likelihood of participants using mHealth interventions,^27^ suggesting that our findings could be explained by the inclusion of relatively mobile participants in the included studies. Of studies reporting EDSS in our review, 16 had a mean participant EDSS of less than 4.5 (equivalent to the ability to walk unaided), with only five reporting a mean EDSS score of 4.5–7.5 (see supplementary materials for more details). Given that ambulation or mobility status is a key component of EDSS and an estimate of appraised disability,^85^ we cannot be sure if CH technologies enabling physical activity-based telerehabilitation are as effective in improving the QoL of people with higher mobility challenges. This is an important area for additional research, given the potential of CH technologies in overcoming existing barriers to accessing in-person support for those with mobility issues, consequent inhibitive transportation or costs,^4^ or those living in rural areas.^86^

In their review, Gromisch and colleagues (2020) reported that mHealth was commonly used by PwMS for symptom management or to improve lifestyle and some psychological outcomes.^27^ Correspondingly, we found CH-mediated self-management interventions to be the most common focus of the studies reviewed, with mixed efficacy tending towards positive impacts on anxiety, depression and QoL. Another systematic review similarly reported promising effects of mHealth-mediated self-assessment and rehabilitation tools on QoL in PwMS, although the strength of evidence was limited given the low number of included studies.^87^ These mixed findings may result from the diverging methodological designs of studies conducted, revealing a need for more rigorous evidence with consistent controls. Additionally, given variability and progression in symptomatology and disability resulting from MS, our findings may not be reflective of how effectively these interventions meet MS sub-type needs, with participants predominantly reporting RRMS. There is a need for comparative studies between MS phenotypes and at different levels of disability, to garner more robust evidence about the effectiveness of CH technologies in delivering interventions to this population.

Our findings also suggest that CH-delivered CBT and cognitive training were more effective than mindfulness-based approaches in improving psychological outcomes and QoL when delivered as a lone intervention. Further, CH-mediated CBT or psychological treatment shows promise in alleviating symptoms of anxiety and depression and enhancing QoL and may be comparable to non-CH-delivered CBT. Websites or web-based programs compared to teleconferencing appear to be more effective.

A systematic review evaluating the influence of online-delivered psychological interventions on psychological outcomes and QoL in PwMS reported similar effectiveness of CBT delivered online.^88^ Converse to our findings, however, they found mindfulness-based interventions equally effective and online-delivered cognitive training less effective in reducing depression and anxiety and enhancing QoL. This may be due to the variety of intervention controls employed across the studies reviewed, which may have redirected effects. These inconsistencies show that while promising, there is a need to further explore and compare the effects of different technologies on specific psychological outcomes.

The paucity of studies on caregivers found is reflective of the limited body of research focusing on MS caregiver needs^20,89,90^ and supportive interventions.^8^ Nonetheless, we found that participating in support groups had positive effects on caregiver anxiety,^38^ mirroring findings of a systematic review exploring the effectiveness of web-delivered peer support on psychological outcomes in caregivers of people with various neurological diseases excluding MS.^91^ Overall, our findings indicate the potential benefit of CH-mediated support groups for caregivers of PwMS, but this requires further evaluation.

An interesting finding was that the efficacy of CH-mediated support group interventions for PwMS varied across studies and outcomes, with some alleviating either anxiety or depression symptoms but not QoL.^62,65,67^ In another systematic review, online peer support for PwMS was seen to provide convenience in emotional support, particularly for those living in rural areas.^92^ There is a potential for CH-mediated peer support to improve psychological wellbeing; however, this requires further evaluation across multiple contexts, particularly comparing rural to urban settings, not highlighted in the studies reviewed.

Findings suggest CH-mediated interventions offer an efficacious solution to improving access for PwMS. Among studies with usual or standard care controls, findings suggest equal efficacy with telerehabilitation,^73,77^ self-management^71^ and CBT-based psychological approaches.^78^ Further, greater efficacy was observed for website-mediated CBT self-management compared to face-to-face CBT,^68^ VR compared to standard cognitive training,^70^ and software-based^72^ or teleconference-mediated education^84^ interventions in improving psychological and/or QoL compared to standard care. Overall, our findings indicate that CH technology has significant potential for the delivery of interventions to improve psychological wellbeing and/or QoL in PwMS and/or their caregivers.

## Strengths and limitations

Our review has a number of strengths. Articles were generally observed to be of high quality, following systematic rigour. We also broadly examined CH technologies rather than focusing on specific types, aggregating evidence gathered during the past decade, a rapid phase of technology adaptation encompassing the COVID-19 pandemic. More importantly, we aggregated evidence in relation to both PwMS and their informal caregivers.

However, though our findings are encouraging, they only suggest possible effectiveness since causation and direction of effects cannot be attributed to specific components of the studies. Equally important, given that the CH technology field is relatively new and rapidly developing, defining its boundaries and delineating overlaps with similar concepts such as telehealth may have impacted article eligibility and inclusion in this review. Furthermore, our criteria excluded studies reported in languages other than English. With the rapid deployment of CH technologies since COVID-19, we might have missed important evaluations of these technologies reported in languages other than English. Additional studies and reports that might have been identified from backward search of citations and grey literature may also have been missed. The included studies not only differed in terms of the design and aims of the interventions, but in the outcomes measured. Even when the same outcomes were measured, different assessments were often used. The combined effect of CH technologies on psychological outcomes could therefore not be computed through a meta-analysis. There are also difficulties in comparing studies that have used different measures of the same construct, despite these measures being validated among PwMS. Thus, there is limited generalizability of findings. This is also noteworthy where no studies disaggregated between urban and rural residence, and the majority were conducted in the USA and Western Europe, making it difficult to interpret its potential to improve access across sub-national or rural/urban locations. Finally, most studies examined psychological outcomes as secondary or incidental outcomes, were primarily focused on improving mobility or fatigue, had varying population sizes and were inconsistent in evaluating the long-term impacts of CH-mediated interventions. Consequently, the conclusions reached in this study require caution and should be considered tentative due to these limitations. These also present an opportunity for further rigorous research, which follows CH concept and practice consensus. Future systematic reviews in this area should update the search to explore recent changes in a rapidly developing area.

## Conclusions

Technology has been widely adopted and adapted to supplement health service provision since the COVID-19 pandemic. Technologies that enable bidirectional communication (CH technologies) have equally been employed in response to barriers limiting access to care. Our review found several pilot interventions since 2019, reflecting increasing demand and interest in these interventions, however few examined long-term effects. There is a need for policies highlighting strategic needs or areas of interest where CH technology can be employed to improve psychological outcomes in PwMS, which would encourage more robust evaluations with appropriate follow-ups to strengthen the evidence base.

The use of CH technologies to deliver interventions shows promise in improving psychological wellbeing and QoL among PwMS and their caregivers. There is, however, a need for rigorous comparative research to generate further evidence for the effectiveness of CH-mediated interventions across different MS types and for informal caregivers. Larger-scale RCTs and qualitative studies will be beneficial to drive the prioritization and scalability of relevant CH-mediated interventions to address psychological wellbeing and QoL in those affected by MS. Likewise, there is a need for development of policies to guide the deployment of CH technologies to improve outcomes for people affected by MS.

## Supplemental Material

sj-docx-1-dhj-10.1177_20552076251326230 - Supplemental material for Impact of connected health on the psychological wellbeing and quality of life of people with multiple sclerosis and their caregivers: A systematic review Supplemental material, sj-docx-1-dhj-10.1177_20552076251326230 for Impact of connected health on the psychological wellbeing and quality of life of people with multiple sclerosis and their caregivers: A systematic review by Joan Alaboson, Laura Coffey, Sowmya Shrivastava, Adeola Ade-Alao and Rebecca Maguire in DIGITAL HEALTH

sj-doc-2-dhj-10.1177_20552076251326230 - Supplemental material for Impact of connected health on the psychological wellbeing and quality of life of people with multiple sclerosis and their caregivers: A systematic review Supplemental material, sj-doc-2-dhj-10.1177_20552076251326230 for Impact of connected health on the psychological wellbeing and quality of life of people with multiple sclerosis and their caregivers: A systematic review by Joan Alaboson, Laura Coffey, Sowmya Shrivastava, Adeola Ade-Alao and Rebecca Maguire in DIGITAL HEALTH

## References

[1] Benito-León J Morales JM Rivera-Navarro J , et al. A review about the impact of multiple sclerosis on health-related quality of life. Disabil Rehabil 2003; 25: 1291–1303. 14617435 10.1080/09638280310001608591

[2] Chiu C Bishop M Pionke JJ , et al. Barriers to the accessibility and continuity of health-care services in people with multiple sclerosis: a literature review. Int J MS Care 2017; 19: 313–321. 29270089 10.7224/1537-2073.2016-016 PMC5734715

[3] Moreno-Torres I Sabin-Munoz J Garcia-Merino A . Multiple sclerosis: epidemiology, genetics, symptoms, and unmet needs. In: Martinez A (eds) Emerging drugs and targets for multiple sclerosis. Royal Soc Chemistry: Cambridge, 2019, pp.3–32.

[4] Gil-González I Martín-Rodríguez A Conrad R , et al. Quality of life in adults with multiple sclerosis: a systematic review. BMJ Open 2020; 10: e041249. 10.1136/bmjopen-2020-041249 PMC7705559 33257490

[5] Ando H Cousins R Young CA . Understanding quality of life across different clinical subtypes of multiple sclerosis: a thematic analysis. Qual Life Res 2022; 31: 2035–2046. DOI: 10.1007/s11136-021-03041-7. 34822047

[6] Fisher PL Salmon P Heffer-Rahn P , et al. Predictors of emotional distress in people with multiple sclerosis: a systematic review of prospective studies. J Affect Disord 2020; 276: 752–764. 32736185 10.1016/j.jad.2020.07.073

[7] Boeschoten RE Braamse AMJ Beekman ATF , et al. Prevalence of depression and anxiety in multiple sclerosis: a systematic review and meta-analysis. J Neurol Sci 2017; 372: 331–341. 20161130. 28017241 10.1016/j.jns.2016.11.067

[8] Rajachandrakumar R Finlayson M . Multiple sclerosis caregiving: a systematic scoping review to map current state of knowledge. Health Soc Care Community 2022; 30: e874–e897. 10.1111/hsc.13687 34935217

[9] Buchanan RJ Huang C . The need for mental health care among informal caregivers assisting people with multiple sclerosis. Int J MS Care 2013; 15: 56–64. 24453764 10.7224/1537-2073.2012-030 PMC3883009

[10] Topcu G Buchanan H Aubeeluck A , et al. Caregiving in multiple sclerosis and quality of life: a meta-synthesis of qualitative research. Psychol Health 2016; 31: 693–710. 20160209. 26742505 10.1080/08870446.2016.1139112

[11] Kobelt G Thompson A Berg J , et al. New insights into the burden and costs of multiple sclerosis in Europe. Multip Scler J 2017; 23: 1123–1136. 10.1177/1352458517694432 PMC5476197 28273775

[12] Finlayson M Cho C . A descriptive profile of caregivers of older adults with MS and the assistance they provide. Disabil Rehabil 2008; 30: 1848–1857. 18608376 10.1080/09638280701707324

[13] Maguire R Maguire P . Caregiver burden in multiple sclerosis: recent trends and future directions. Curr Neurol Neurosci Rep 2020; 20: 18. 32444986 10.1007/s11910-020-01043-5 PMC7242779

[14] Di Cara M Grezzo D Palmeri R , et al. Psychological well-being in people with multiple sclerosis: a descriptive review of the effects obtained with mindfulness interventions. Neurol Sci 2022; 43: 211–217. 34697659 10.1007/s10072-021-05686-1 PMC8724219

[15] Beer S Khan F Kesselring J . Rehabilitation interventions in multiple sclerosis: an overview. J Neurol 2012; 259: 1994–2008. 22772357 10.1007/s00415-012-6577-4

[16] Petrin J Donnelly C McColl MA , et al. Is it worth it?: the experiences of persons with multiple sclerosis as they access health care to manage their condition. Health Expect 2020; 23: 1269–1279. 33145866 10.1111/hex.13109 PMC7696118

[17] Golinelli D Boetto E Carullo G , et al. Adoption of digital technologies in health care during the COVID-19 pandemic: systematic review of early scientific literature. J Med Internet Res 2020; 22: e22280. 10.2196/22280 PMC7652596 33079693

[18] Manacorda T Bandiera P Terzuoli F , et al. Impact of the COVID-19 pandemic on persons with multiple sclerosis: early findings from a survey on disruptions in care and self-reported outcomes. J Health Serv Res Policy 2021; 26: 189–197. 33337256 10.1177/1355819620975069 PMC8182334

[19] Kinyanjui B McDaniels B Frain M , et al. Healthcare and Rehabilitation Needs of Individuals with Multiple Sclerosis. Contemp Res Disabil Rehabil 2018; 1. DOI: 10.51734/crdr.v1i1.29.

[20] Appleton D Robertson N Mitchell L , et al. Our disease: a qualitative meta-synthesis of the experiences of spousal/partner caregivers of people with multiple sclerosis. Scand J Caring Sci 2018; 32: 1262–1278. 30144143 10.1111/scs.12601

[21] Pattichis CS Panayides AS . Connected Health. Front Digit Health 2019; 1: 1. 20191016. DOI: 10.3389/fdgth.2019.00001. 34713013 PMC8519500

[22] Caulfield BM Donnelly SC . What is connected health and why will it change your practice? Qjm 2013; 106: 703–707. 23676416 10.1093/qjmed/hct114

[23] Chen SC Liu C Hu R , et al. Nomen omen": exploring connected healthcare through the perspective of name omen. Healthcare (Basel) 2020; 8: 20200323. 10.3390/healthcare8010066 PMC7151183 32210024

[24] Scholz M Haase R Schriefer D , et al. Electronic health interventions in the case of multiple sclerosis: from theory to practice. Brain Sci 2021; 11: 180. 33540640 10.3390/brainsci11020180 PMC7913051

[25] Heesen C Berger T Riemann-Lorenz K , et al. Mobile health interventions in multiple sclerosis: a systematic review. Multiple Sclerosis Journal 2023; 29: 1709–1720. 37897326 10.1177/13524585231201089 PMC10687804

[26] Cohen M . Connected health and multiple sclerosis. Rev Neurol (Paris) 2018; 174: 480–485. 29680178 10.1016/j.neurol.2018.03.008

[27] Gromisch ES Turner AP Haselkorn JK , et al. Mobile health (mHealth) usage, barriers, and technological considerations in persons with multiple sclerosis: a literature review. JAMIA Open 2020; 4: ooaa067. DOI: 10.1093/jamiaopen/ooaa067. PMC8423420 34514349

[28] Page MJ McKenzie JE Bossuyt PM , et al. The PRISMA 2020 statement: an updated guideline for reporting systematic reviews. Syst Rev 2021; 10: 89. 33781348 10.1186/s13643-021-01626-4 PMC8008539

[29] Del-Pino-Casado R Priego-Cubero E López-Martínez C , et al. Subjective caregiver burden and anxiety in informal caregivers: a systematic review and meta-analysis. PLOS ONE 2021; 16: e0247143. 10.1371/journal.pone.0247143 PMC7920375 33647035

[30] Fraser MJ Gorely T O’Malley C , et al. Does connected health technology improve health-related outcomes in rural cardiac populations? Systematic review narrative synthesis. Int J Environ Res Public Health 2022; 19: 2302. 35206493 10.3390/ijerph19042302 PMC8871734

[31] Haraldstad K Wahl A Andenæs R , et al. A systematic review of quality of life research in medicine and health sciences. Qual Life Res 2019; 28: 2641–2650. 31187410 10.1007/s11136-019-02214-9 PMC6761255

[32] Li Z-S Hasson F . Resilience, stress, and psychological well-being in nursing students: a systematic review. Nurse Educ Today 2020; 90: 104440. 32353643 10.1016/j.nedt.2020.104440

[33] Hopewell S McDonald S Clarke MJ , et al. Grey literature in meta-analyses of randomized trials of health care interventions. Cochrane Database Syst Rev 2007; 2007: MR000010. 10.1002/14651858.MR000010.pub3 PMC8973936 17443631

[34] Haddaway NR Collins AM Coughlin D , et al. The role of Google Scholar in evidence reviews and its applicability to grey literature searching. PLOS ONE 2015; 10: e0138237. 10.1371/journal.pone.0138237 PMC4574933 26379270

[35] Ouzzani M Hammady H Fedorowicz Z , et al. Rayyan—a web and mobile app for systematic reviews. Syst Rev 2016; 5: 10. 27919275 10.1186/s13643-016-0384-4 PMC5139140

[36] Hong QN Fàbregues S Bartlett G , et al. The mixed methods appraisal tool (MMAT) version 2018 for information professionals and researchers. Educ Inf 2018; 34: 285–291.

[37] Dixon-Woods M Agarwal S Jones D , et al. Synthesising qualitative and quantitative evidence: a review of possible methods. J Health Serv Res Policy 2005; 10: 45–53. 15667704 10.1177/135581960501000110

[38] Khazaeili M Hajebi MZ Mohamadkhani P , et al. The effectiveness of mindfulness-based intervention on anxiety, depression and burden of caregivers of multiple sclerosis patients through web conferencing. J Pract Clin Psychology 2019; 7: 21–32.

[39] Claflin SB Mainsbridge C Campbell J , et al. Self-reported behaviour change among multiple sclerosis community members and interested laypeople following participation in a free online course about multiple sclerosis. Health Promot J Aust 2022; 33: 768–778. 10.1002/hpja.559 34807490

[40] Halstead EJ Leavitt VM Fiore D , et al. A feasibility study of a manualized resilience-based telehealth program for persons with multiple sclerosis and their support partners. Mult Scler J Exp Transl Clin 2020; 6: 2055217320941250. DOI: 10.1177/2055217320941250. 32913660 PMC7444140

[41] Bessing B van der Mei I Taylor BV , et al. Evaluating the impact of the Understanding Multiple Sclerosis online course on participant MS knowledge, health literacy, resilience, self-efficacy, quality of life, and MS symptom severity. Mult Scler Relat Disord 2022; 60: 103717. DOI: 10.1016/j.msard.2022.103717. 35259682

[42] Kahraman T Savci S Ozdogar AT , et al. Physical, cognitive and psychosocial effects of telerehabilitation-based motor imagery training in people with multiple sclerosis: a randomized controlled pilot trial. J Telemed Telecare 2020; 26: 251–260. 30744491 10.1177/1357633X18822355

[43] Pratap A Grant D Vegesna A , et al. Evaluating the utility of smartphone-based sensor assessments in persons with multiple sclerosis in the real-world using an app (elevateMS): observational, prospective pilot digital health study. JMIR Mhealth Uhealth 2020; 8: e22108. 10.2196/22108 PMC7655470 33107827

[44] Alschuler KN Arewasikporn A Nelson IK , et al. Promoting resilience in individuals aging with multiple sclerosis: results from a pilot randomized controlled trial. Rehabil Psychol 2018; 63: 338–348. 30024203 10.1037/rep0000223

[45] Boeschoten RE Nieuwenhuis MM van Oppen P , et al. Feasibility and outcome of a web-based self-help intervention for depressive symptoms in patients with multiple sclerosis: a pilot study. J Neurol Sci 2012; 315: 104–109. 22133479 10.1016/j.jns.2011.11.016

[46] Bogosian A Chadwick P Windgassen S , et al. Distress improves after mindfulness training for progressive MS: a pilot randomised trial. Multip Scler (Houndmills, Basingstoke, England) 2015; 21: 1184–1194. 10.1177/1352458515576261 25767124

[47] Bulbul SB Keser I Yucesan C , et al. Effects of pelvic floor muscle training applied with telerehabilitation in patients with multiple sclerosis having lower urinary track symptoms: a randomized controlled trial. Health Care Women Int 2024; 45: 731–747. DOI: 10.1080/07399332.2023.2190593. 37010419

[48] Cavalera C Rovaris M Mendozzi L , et al. Online meditation training for people with multiple sclerosis: a randomized controlled trial. Multip Scler J 2019; 25: 610–617. 10.1177/1352458518761187 29485319

[49] Chen MH Cherian C Elenjickal K , et al. Real-time associations among MS symptoms and cognitive dysfunction using ecological momentary assessment. Front Med (Lausanne) 2023; 9: 1049686. DOI: 10.3389/fmed.2022.1049686. 36714150 PMC9877417

[50] Chikersal P Venkatesh S Masown K , et al. Predicting multiple sclerosis outcomes during the COVID-19 stay-at-home period: observational study using passively sensed behaviors and digital phenotyping. JMIR Ment Health 2022; 9: e38495. DOI: 10.2196/38495. PMC9407162 35849686

[51] Claflin SB Campbell J Taylor BV . Healthcare utilisation and perceived healthcare accessibility and quality amongst people living with multiple sclerosis enroled in an online course. Mult Scler Relat Disord 2023; 73: 104621. 36965220 10.1016/j.msard.2023.104621

[52] Dogru-Huzmeli E Duman T Cakmak AI , et al. Can diplopia complaint be reduced by telerehabilitation in multiple sclerosis patient during the pandemic?: a case report. Neurol Sci 2021; 42: 4387–4390. 33763810 10.1007/s10072-021-05194-2 PMC7990493

[53] Donkers SJ Nickel D Paul L , et al. Adherence to physiotherapy-guided web-based exercise for persons with moderate-to-severe multiple sclerosis: a randomized controlled pilot study. Int J MS Care 2020; 22: 208–214. 33177956 10.7224/1537-2073.2019-048 PMC7643843

[54] Dunne J Chih HJ Begley A , et al. A randomised controlled trial to test the feasibility of online mindfulness programs for people with multiple sclerosis. Mult Scler Relat Disord 2021; 48: 102728. DOI: 10.1016/j.msard.2020.102728. 33477003

[55] Fischer A Schroder J Vettorazzi E , et al. An online programme to reduce depression in patients with multiple sclerosis: a randomised controlled trial. Lancet Psychiatry 2015; 2: 217–223. 26359900 10.1016/S2215-0366(14)00049-2

[56] Flachenecker P Bures AK Gawlik A , et al. Efficacy of an internet-based program to promote physical activity and exercise after inpatient rehabilitation in persons with multiple sclerosis: a randomized, single-blind, controlled study. Int J Environ Res Public Health 2020; 17. DOI: 10.3390/ijerph17124544. PMC7344392 32599767

[57] Gandy M Heriseanu AI Balakumar T , et al. The wellbeing neuro course: a randomised controlled trial of an internet-delivered transdiagnostic psychological intervention for adults with neurological disorders. Psychol Med 2023; 53: 6817–6827. DOI: 10.1017/S0033291723000338. 39625264 PMC10600819

[58] Gandy M Karin E McDonald S , et al. A feasibility trial of an internet-delivered psychological intervention to manage mental health and functional outcomes in neurological disorders. J Psychosom Res 2020; 136: 110173. DOI: 10.1016/j.jpsychores.2020.110173. 32623193

[59] Golan D Sagiv S Glass-Marmor L , et al. Mobile-phone-based e-diary derived patient reported outcomes: association with clinical disease activity, psychological status and quality of life of patients with multiple sclerosis. PLoS One 2021; 16: e0250647. 10.1371/journal.pone.0250647 PMC8099126 33951061

[60] Jeong IC Karpatkin H Finkelstein J . Physical telerehabilitation improves quality of life in patients with multiple sclerosis. Stud Health Technol Inform 2021; 284: 384–388. 34920553 10.3233/SHTI210752

[61] Jongen PJ Sinnige LG van Geel BM , et al. The interactive web-based program MSmonitor for self-management and multidisciplinary care in multiple sclerosis: concept, content, and pilot results. Patient Prefer Adherence 2015; 9: 1741–1750. 26715841 10.2147/PPA.S93783 PMC4685885

[62] Kever A Aguerre IM Vargas W , et al. Feasibility trial of a telehealth support group intervention to reduce anxiety in multiple sclerosis. Clin Rehabil 2022; 36: 1305–1313. 35673256 10.1177/02692155221107077

[63] Kratz AL Alschuler KN Williams DA , et al. Development and pilot testing of a web-based symptom management program for multiple sclerosis: my MS toolkit. Rehabil Psychol 2021; 66: 224–232. 33539138 10.1037/rep0000375

[64] Landtblom AM Guala D Martin C , et al. Rebiqol: a randomized trial of telemedicine patient support program for health-related quality of life and adherence in people with MS treated with rebif. PLoS One 2019; 14: e0218453. 10.1371/journal.pone.0218453 PMC6611587 31276502

[65] Leavitt VM Riley CS De Jager PL , et al. Esupport: feasibility trial of telehealth support group participation to reduce loneliness in multiple sclerosis. Multiple Sclerosis Journal 2020; 26: 1797–1800. 31668134 10.1177/1352458519884241

[66] Limmroth V Bayer-Gersmann K Mueller C , et al. Ascertaining medication use and patient-reported outcomes via an app and exploring gamification in patients with multiple sclerosis treated with interferon beta-1b: observational study. JMIR Format Res 2022; 6: e31972. DOI: 10.2196/31972. PMC8929528 35285806

[67] McArthur AR Peterson EW Sosnoff J , et al. Online delivery of the individualized reduction of falls intervention for persons with multiple sclerosis who use a wheelchair or scooter full-time: a pilot study. Int J MS Care 2023; 25: 82–90. 36923574 10.7224/1537-2073.2022-044 PMC10010107

[68] Moss-Morris R McCrone P Yardley L , et al. A pilot randomised controlled trial of an internet-based cognitive behavioural therapy self-management programme (MS Invigor8) for multiple sclerosis fatigue. Behav Res Ther 2012; 50: 415–421. 22516321 10.1016/j.brat.2012.03.001

[69] Najafi P Hadizadeh M Cheong JPG , et al. Effects of tele-pilates and tele-yoga on biochemicals, physical, and psychological parameters of females with multiple sclerosis. J Clin Med 2023; 12: 1585. DOI: 10.3390/jcm12041585. 36836119 PMC9966519

[70] Pagliari C Di Tella S Jonsdottir J , et al. Effects of home-based virtual reality telerehabilitation system in people with multiple sclerosis: A randomized controlled trial. J Telemed Telecare 2024; 30: 344–355. DOI: 10.1177/1357633X211054839. 34851211

[71] Paul L Coulter EH Miller L , et al. Web-based physiotherapy for people moderately affected with multiple sclerosis; quantitative and qualitative data from a randomized, controlled pilot study. Clin Rehabil 2014; 28: 924–935. 24691218 10.1177/0269215514527995

[72] Pottgen J Friede T Lau S , et al. Managing neuropsychological impairment in multiple sclerosis: Controlled study on a standardized metacognitive intervention (MaTiMS). Mult Scler Relat Disord 2022; 59: 103687. DOI: 10.1016/j.msard.2022.103687. 35189580

[73] Sadeghi N Eelen P Nagels G , et al. Innovating care in multiple sclerosis: feasibility of synchronous internet-based teleconsultation for longitudinal clinical monitoring. J Pers Med 2022; 12: 433. DOI: 10.3390/jpm12030433. 35330433 PMC8948780

[74] Saladino ML Gualtieri C Scaffa M , et al. Neuro rehabilitation effectiveness based on virtual reality and tele rehabilitation in people with multiple sclerosis in Argentina: Reavitelem study. Mult Scler Relat Disord 2023; 70: 104499. DOI: 10.1016/j.msard.2023.104499. 36645996

[75] Sangelaji B Smith C Paul L , et al. Promoting physical activity engagement for people with multiple sclerosis living in rural settings: a proof-of-concept case study. Eur J Physiother 2017; 19: 17–21.

[76] Sesel AL Sharpe L Beadnall HN , et al. A randomized controlled trial of a web-based mindfulness programme for people with MS with and without a history of recurrent depression. Multip Scler J 2022; 28: 1392–1401. 10.1177/13524585211068002 35130768

[77] Tarakci E Tarakci D Hajebrahimi F , et al. Supervised exercises versus telerehabilitation. Benefits for persons with multiple sclerosis. Acta Neurol Scand 2021; 144: 303–311. 33961295 10.1111/ane.13448

[78] Turkowitch D Ludwig R Nelson E , et al. Telehealth-delivered cognitive behavioral therapy for insomnia in individuals with multiple sclerosis: a pilot study. Mult Scler Int 2022; 2022: 7110582. DOI: 10.1155/2022/7110582. 35281348 PMC8906967

[79] Turner AP Hartoonian N Sloan AP , et al. Improving fatigue and depression in individuals with multiple sclerosis using telephone-administered physical activity counseling. J Consult Clin Psychol 2016; 84: 297–309. 26913621 10.1037/ccp0000086

[80] Van Beek JJW Lehnick D Pastore-Wapp M , et al. Tablet app-based dexterity training in multiple sclerosis (TAD-MS): a randomized controlled trial. Disabil Rehabil-Assist Technol 2024; 19: 889–899. DOI: 10.1080/17483107.2022.2131915. 36308305

[81] Van Geel F Geurts E Abasiyanik Z , et al. Feasibility study of a 10-week community -based program using the WalkWithMe application on physical activity, walking, fatigue and cognition in persons with Multiple Sclerosis. Mult Scler Relat Disord 2020; 42: 102067. DOI: 10.1016/j.msard.2020.102067. 32371377

[82] Van Kessel K Wouldes T Moss-Morris R . A New Zealand pilot randomized controlled trial of a web-based interactive self-management programme (MSInvigor8) with and without email support for the treatment of multiple sclerosis fatigue. Clin Rehabil 2016; 30: 454–462. 25952587 10.1177/0269215515584800

[83] Wingo BC Rinker JR Goss AM , et al. Feasibility of improving dietary quality using a telehealth lifestyle intervention for adults with multiple sclerosis. Mult Scler Relat Disord 2020; 46: 102504. DOI: 10.1016/j.msard.2020.102504. 32942117

[84] Zissman K Lejbkowicz I Miller A . Telemedicine for multiple sclerosis patients: assessment using health value compass. Multiple Sclerosis Journal 2012; 18: 472–480. 21965420 10.1177/1352458511421918

[85] Kurtzke JF . Rating neurologic impairment in multiple sclerosis. An Expanded Disability status Scale (EDSS) 1983; 33: 1444–1444. 10.1212/wnl.33.11.1444 6685237

[86] Zhou L Parmanto B . Reaching people with disabilities in underserved areas through digital interventions: systematic review. J Med Internet Res 2019; 21: e12981. 10.2196/12981 PMC7380899 31654569

[87] Bonnechère B Rintala A Spooren A , et al. Is mHealth a useful tool for self-assessment and rehabilitation of people with multiple sclerosis? A systematic review. Brain Sci 2021; 11: 1187. 34573208 10.3390/brainsci11091187 PMC8466296

[88] Montañés-Masias B Bort-Roig J Pascual JC , et al. Online psychological interventions to improve symptoms in multiple sclerosis: a systematic review. Acta Neurol Scand 2022; 146: 448–464. 36121184 10.1111/ane.13709 PMC9825977

[89] Corry M While A . The needs of carers of people with multiple sclerosis: a literature review. Scand J Caring Sci 2009; 23: 569–588. 19077062 10.1111/j.1471-6712.2008.00645.x

[90] McKeown LP Porter-Armstrong AP Baxter GD . The needs and experiences of caregivers of individuals with multiple sclerosis: a systematic review. Clin Rehabil 2003; 17: 234–248. 12735530 10.1191/0269215503cr618oa

[91] Wallace SJ Kothari J Jayasekera A , et al. Do caregivers who connect online have better outcomes? A systematic review of online peer-support interventions for caregivers of people with stroke, dementia, traumatic brain injury, Parkinson’s disease and multiple sclerosis. Brain Impair 2021; 22: 233–259.

[92] Gerritzen EV Lee AR McDermott O , et al. Online peer support for people with multiple sclerosis: a narrative synthesis systematic review. Int J MS Care 2022; 24: 252–259. 36545647 10.7224/1537-2073.2022-040 PMC9749829

