# Supplementary material for: Impact of connected health on the psychological wellbeing and quality of life of people with multiple sclerosis and their caregivers: A systematic review
Source: Digit Health. 2025 Mar 31;11:20552076251326230. doi: 10.1177/20552076251326230 (PMC11960181; doi:10.1177/20552076251326230)
Supplement: sj-doc-2-dhj-10.1177_20552076251326230 - Supplemental material for Impact of connected health on the psychological wellbeing and quality of life of people with multiple sclerosis and their caregivers: A systematic review [file sj-doc-2-dhj-10.1177_20552076251326230.doc]

**Supplementary material 1: Search Strategy: definition of concepts, keywords, search logic and search results.**

**Concept definition**

*Psychological wellbeing*

Psychological wellbeing relates to self-reported positive and negative constructs of wellbeing or distress shaped by personal experiences and aspects of life such as fear, sadness, happiness or satisfaction

*Quality of life*

Subjective appraisal or evaluation of one’s life, with emphasis on perceived health status and activity limitation.

*Connected health technology*

A connected health intervention can include any digital health, ehealth, mhealth, web-based health or other internet-facilitated intervention that features user (people with Ms [PwMS] and/or their informal caregivers) input, processing and/or feedback. In order to be considered ‘connected’, the technology must offer a two-way flow of information

*Caregivers*

A caregiver is any person providing support (physical, emotional or social) outside healthcare-professionals at home or within the community

**Search logic**

A (connected health) AND B (wellbeing) AND C (caregivers or patients) AND D (MS)

Keywords

| **A**  **“**Connected health” OR eHealth OR mHealth OR m-health OR “digital health” OR “mobile health” OR Telehealth OR “Electronic Health Record” OR “electronic medical record” OR App OR Web OR Online OR wearabl* OR “Electronic health services” OR Telemedicine OR Smartphone OR “mobile application” OR telemedicine"[MeSH Terms] OR “Smartphone"[MeSH Terms] OR "mobile applications"[MeSH Terms] | **B**  anxiety OR depression OR “psychological distress” OR “mental health” OR “psychological well-being” OR “Psychological wellbeing” OR sadness OR fear OR “life satisfaction” OR “personal satisfaction” OR “emotional wellbeing” OR “emotional well-being” OR “Quality of life” OR  “Health-related quality of life“ OR QOL MESH OR ANXIETY MESH OR DEPRESSION MESH |
| --- | --- |
| **C**  Patient* OR People OR OR Adults OR PwMS OR perso* OR caregiv* OR carer* OR relatives OR informal OR kin OR famil* OR Parent OR Sibling OR Partner OR Spous* OR Friend OR “home support” OR “community support” | **D**  “multiple sclerosis” OR MS OR “Primary Progressive Multiple sclerosis” OR “secondary Progressive Multiple sclerosis” OR “remitting relapsing multiple sclerosis” OR Multiple sclerosis MESH |

Search results

| **Database and date of search** | **Search string** | **All results** | **Limiter: 2012 -2023** |
| --- | --- | --- | --- |
| **PubMed**  19/04/2023 | Search: (((((((((((((((((((((("Connected health"[Title/Abstract]) OR (eHealth[Title/Abstract])) OR (mHealth[Title/Abstract])) OR (m-health[Title/Abstract])) OR ("mobile health"[Title/Abstract])) OR ("digital health"[Title/Abstract])) OR (Telehealth[Title/Abstract])) OR ("Electronic Health Record"[Title/Abstract])) OR ("electronic medical record"[Title/Abstract])) OR (App[Title/Abstract])) OR (Web[Title/Abstract])) OR (Online[Title/Abstract])) OR (wearabl*[Title/Abstract])) OR ("Electronic health services"[Title/Abstract])) OR (telemedicine[Title/Abstract])) OR (smartphone[Title/Abstract])) OR (mobile applications[Title/Abstract])) OR ("Telemedicine"[Mesh])) OR ("Smartphone"[Mesh])) OR ("Mobile Applications"[Mesh])) AND (((((((((((((((((anxiety[Title/Abstract]) OR (depression[Title/Abstract])) OR ("psychological distress"[Title/Abstract])) OR ("mental health"[Title/Abstract])) OR ("psychological well-being"[Title/Abstract])) OR ("psychological wellbeing"[Title/Abstract])) OR (sadness[Title/Abstract])) OR (fear[Title/Abstract])) OR ("life satisfaction"[Title/Abstract])) OR ("personal satisfaction"[Title/Abstract])) OR ("emotional wellbeing"[Title/Abstract])) OR ("emotional well-being"[Title/Abstract])) OR ("Quality of life"[Title/Abstract])) OR ("Health related quality of life"[Title/Abstract])) OR ("Quality of Life"[Mesh])) OR ("Anxiety"[Mesh])) OR ("Depression"[Mesh]))) AND ((((((((((((((((((Patient*[Title/Abstract]) OR (people[Title/Abstract])) OR (adults[Title/Abstract])) OR (PwMS[Title/Abstract])) OR (perso*[Title/Abstract])) OR (caregiv*[Title/Abstract])) OR (carer*[Title/Abstract])) OR (relatives[Title/Abstract])) OR (informal[Title/Abstract])) OR (kin[Title/Abstract])) OR (famil*[Title/Abstract])) OR (Parent[Title/Abstract])) OR (Partner[Title/Abstract])) OR (Spous*[Title/Abstract])) OR (Friend[Title/Abstract])) OR ("home support"[Title/Abstract])) OR ("community support"[Title/Abstract])) OR (Sibling[Title/Abstract]))) AND (((((("multiple sclerosis"[Title/Abstract]) OR (MS[Title/Abstract])) OR ("Primary Progressive Multiple sclerosis"[Title/Abstract])) OR ("secondary Progressive Multiple sclerosis"[Title/Abstract])) OR ("remitting relapsing multiple sclerosis"[Title/Abstract])) OR ("Multiple Sclerosis"[Mesh])) | 592 | 568 |
| **PsycINFO**  19/04/2023 | S1 AND S2 AND S3 AND S4  (See attachment) | 171 | 160 |
| **Web of Science**  19/04/2023 | #1 AND #2 AND #3 AND #4 (see attachment) | 801 | 762 |
| **Embase**  (19/04/2023 | #1 AND #2 AND #3 AND #4 (see attachment) | 1203 | 1133 |
| **CINAHYL** 28/04/2023 | #1 AND #2 AND #3 AND #4 (see attachment) | 215 | 198 |

**Supplementary material 2: Eligibility criteria description**

|  | **Inclusionary Criteria** | **Exclusionary Criteria** |
| --- | --- | --- |
| **Population** | - Adults with multiple sclerosis (MS) and or caregivers of person with MS (PwMS) with no restriction on type, severity, or disability status. - An adult is defined as an individual aged 18 years or above. - Separate analysis of adult MS participants where MS is part of a larger population including children and or other diseases being studied | - PwMS below 18 years - People with MS below 18 years included and no separate analysis done for adults |
| I**ntervention** | - Relevant to studies evaluating any connected health technology intervention that includes smartphones, web‐based interventions, apps, electronic medical/health records, online group‐based interventions, telehealth, telemedicine, and wearables, mobile application - No restrictions on the timing of the intervention as long as the intervention is on people with MS or their caregivers with a confirmed MS diagnosis. | - Interventions that are not connected to the internet or not CH technology being studied. - Not evaluating any impact or effect of CH on psychological outcomes described below(e.g., just a descriptive and no aims to evaluate the impact of the intervention) |
| **Measures** | - Primary data from the PwMS and or their caregivers using validated measures (in relation to quantitative studies) | Studies with the following characteristics of measures:   - Secondary data - Using unvalidated measures - CH interventions without a measure of psychological outcomes or quality of life. - Studies using no measure |
| **Outcomes** | - Psychological outcomes or quality of life below   Psychological wellbeing: ppsychological wellbeing relates to self-reported positive and negative constructs of wellbeing or distress shaped by personal experiences and aspects of life {e.g., anxiety, depression, fear, sadness, panic, happiness and satisfaction}  Quality of life: subjective appraisal or evaluation of one’s life, with emphasis on perceived health status and activity limitation. | - Study outcomes are unrelated to psychological wellbeing or quality of life. |
| **Study Design** | - All study designs as long as there is some evaluation of CH intervention and the effect of the intervention on psychological outcomes or quality of life was reported. | - Literature reviews, review of reviews, systematic reviews, meta-analysis, background articles, commentaries, descriptive designs, cost-effectiveness studies, or study protocols - Any ongoing trial which is not completed nor published yet |
| **Reporting** | - Reports in the English language - Article appeared in a peer-reviewed journal - Published within the past 10 years. | - Reports in languages other than English - Grey literature such as thesis and reports - Studies published prior to 2012. |

**Supplementary material 3:** Study methodology, intervention duration, summary outcome, and quality of included article

| **Author** | **Study design** | **Type of connected health technology** | **Population** | **Intervention characteristics** | **Intervention duration in weeks** | **Instruments (validated tools) used** | **Comparator** | **Outcome** | **MMAT quality assessment** |
| --- | --- | --- | --- | --- | --- | --- | --- | --- | --- |
| Alschuler et al ^1^ | A single centre two-group pilot RCT | Group tele-conference | PwMS | Psychoeducation and skills with positive psychology training | 6 | Quality of Life in Neurological Disorders (Neuro-QoL); PROMIS Anxiety Short Answer; Subjective happiness scale | Wait-list control | No significant improvement in positive affect, wellbeing, anxiety, happiness and depression in intervention group compared to control | High -4 |
| Bessing et al ^2^ | Cohort study | Massive open online course (MOOC) | PwMS  People without MS | Course providing knowledge and information about MS | 6 | Personal wellbeing index | n/a | No change in QoL in PwMS but some improvement in QoL of people without MS | Moderate -3 |
| Boeschoten et al ^3^ | Pre-post study | Web-based | PwMS | Psychoeducation; self-management support | 5 | Beck depression inventory- II (BDI); Anxiety subscale of the Hospital Anxiety and Depression Scale (HADS); EuroQol quality of life measure (EQ-5D-5L) | n/a | Lower depression and anxiety symptoms in intervention group completers than non-completers including in those with diagnosis of major depression or anxiety disorder. Significant difference in psychological impact between groups. | High - 4 |
| Bogosian et al ^4^ | Parallel-group, RCT | Teleconference | PwMS | Mindfulness based cognitive therapy | 8 | General Health Questionnaire (GHQ-12); HADS;  Multiple Sclerosis Impact Scale (MSIS-29) | Wait list control | Lower psychological distress in intervention than control group at intervention and 3 months follow-up. Lower anxiety significant post-intervention but not at 3 months follow-up. No significant difference in depression. | High -5 |
| Bulbul et al ^5^ | RCT | Video telerehabilitation | PwMS | Telerehabilitation of pelvic floor muscle training; feedback support | 8 | Kings Health Questionnaire (KHQ) | Lifestyle only advice control | Significant improvement in QoL in intervention group compared to control group | High - 4 |
| Cavalera et al ^6^ | RCT | Video conference - skype; website | PwMS | Group psychoeducation course | 8 | Multiple Sclerosis Quality of Life-54 (MSQoL-54);  HADS | Active control: Online psychoeducation | Significant improvement in QoL and lower anxiety and depression following intervention compared control group. But no difference between groups at 6-month follow-up | High - 5 |
| Chen et al ^7^ | Cohort study | Study app; video conferencing | PwMS | Symptom monitoring | 3 | Ecological momentary Assessment | n/a | Significant improvement in anxiety but not in depression during invention | High - 5 |
| Chikersal et al ^8^ | Natural experiment: | Mobile app; Fitbit Inspire HR | PwMS | Remote monitoring | 12 | PHQ-9 | n/a | Improvement in depression | High - 5 |
| Claflin et al ^9^ | Longitudinal cohort study | MOOC | PwMS | Education; information support | 6 | Personal wellbeing index | n/a | Improvement in QoL following intervention | Moderate - 3 |
| Claflin et al ^10^ | Observational cohort study | MOOC | PwMS; Caregivers | Education | 6 | Personal wellbeing index;  Brief Resilience Scale | n/a | No difference in HRQoL  between baseline and intervention | Moderate - 3 |
| Dogru-Huzmeli et al ^11^ | Case report | Telerehabilitation using WhatsApp video | PwMS | Telerehabilitation - Cawthorne Cooksey exercises | 10 | Short Form-36 (SF-36) | n/a | Improvement in participants’ QoL post-rehabilitation | High - 4 |
| Donkers et al ^12^ | Single-blinded RCT | Website | PwMS | Telephysiotherapy exercises | 26 | MSIS-29; HADS | Active comparator: usual care exercise | Medium effects seen in mean improvement in anxiety, depression and psychological impact in intervention compared to control group | High - 5 |
| Dunne et al ^13^ | Mixed-methods embedded within an RCT | Teleconferencing - Zoom | PwMS | Online mindfulness programs | 8 | MSQoL-54 | Wait-list control | No significant effect on QoL or wellbeing in intervention group compared to control | High - 4 |
| Fischer et al ^14^ | RCT | Online /web-based | PwMS | Tele-CBT; education | 9 | BDI;  WHO Quality of Life scale (WHO-QoL BREF); Hamburg Quality of Life  Questionnaire for Multiple Sclerosis.  (HAQUAMS) | Waitlist control | Improvement in depression in the intervention group. Only the psychological wellbeing aspect of QoL improved in the intervention group compared to control | High - 4 |
| Flachenecker et al ^15^ | RCT | Internet-based software | PwMS | Tele-exercise and physical activity; Diary progress tracking | 12 | MSIS-29 | Control | Improvement in HRQoL after intervention in intervention and control but remained elevated in intervention group alone at 3- and 6-month follow-up | High - 4 |
| Gandy et al ^16^ | RCT | Website and email (or telephone) support | PwMS | Online psycho-education - CBT; self-management | 10 | PHQ-9;  GAD-7 | Control | Significant improvements in depression and anxiety symptoms in intervention group compared to control | Moderate -3 |
| Gandy et al ^17^ | Single-group open-trial | Website | PwMS | Tele-CBT; education and cognitive rehabilitation | 6 | PHQ-9; Generalised Anxiety Disorder Scale 7-Item (GAD-7) | n/a | Significant improvement in depression and anxiety following intervention in participants at 6-week and 3-month follow-up | High - 4 |
| Golan et al ^18^ | Cohort study. | Smartphone app | PwMS | Diary for symptom monitoring; monthly reporting | 52 | abbreviated version of the generic world health organization quality of life questionnaire (WHOQOL-BREF);  HADS | n/a | Negative association with intervention seen for anxiety. Significant positive QoL changes following intervention | High - 4 |
| Halstead et al ^19^ | Cohort study | Web-based portal and or software | PwMS; Caregivers | Psychoeducation and practical skills | 6 | HADS;  General Life satisfaction Survey; Positive and Negative Affect Schedule | n/a | Improvement in life satisfaction, increased positive emotion along with lower negative emotions reported. Lower anxiety in both caregivers and PwMS | High - 5 |
| Jeong et al ^20^ | RCT | Home-based telerehabilitation | PwMS | Exercise telerehabilitation; self-management | 12 | MSQOL-54 | Control group | Mean difference of some improvement in aspects of QoL in intervention than control group between baseline and 3-month follow-up | High - 4 |
| Jongen et al ^21^ | Pre-post study | Web-based program | PwMS | Self-management support | 312 | Leeds Multiple Sclerosis Quality of Life (LMSQoL); | n/a | Improved average HRQoL after intervention | High -4 |
| Kahraman et al ^22^ | RCT | Videoconferencing using Skype | PwMS;  People without MS | Telerehabilitation-based motor imaging training (Tele-MIT) | 8 | HADS; Multiple Sclerosis International Quality of Life questionnaire (MusiQoL) | Control with MS and people without MS | Intervention group showed improvements with lower anxiety and depression and higher QoL than both control. | High - 5 |
| Kever et al ^23^ | A phase I and II non-randomized clinical feasibility trial | Online support intervention | PwMS | Support group | 12 | (normalized) total state anxiety score on the State-Trait Anxiety Scale (STAI); 44-item Functional Assessment of Multiple Sclerosis, (FAMS); PHQ-9 | No treatment control | Lower anxiety in intervention group, but no between-group difference in QoL and depression seen. | High - 4 |
| Khazaeili et al ^24^ | Pre and post-test quasi-experimental study with control group | Web conferencing - Telegram | Caregivers | Remote mindfulness-based cognitive therapy and stress therapy; social media support group | 8 | BDI | No intervention control | Significantly lower anxiety but no difference in depression in intervention group compared to control. | Low -1 |
| Kratz et al ^25^ | Single-arm pilot trial | Web-based multiple-symptom self-management program | PwMS | Symptom self-management; Tele-CBT | 12 | PHQ-9 | n/a | No significant improvement in depression observed after intervention | High - 5 |
| Landtblom et al ^26^ | Randomized, comparative, multicentre study | Emails providing information; Website | PwMS | Remote monitoring; information support; diary for progress monitoring | 52 | MSIS-29;  EQ-5D-5L | Technical support | Intervention group showed no significant difference in HRQoL | Moderate -3 |
| Leavitt et al ^27^ | Single-blind pilot RCT | Online support group | PwMS | Support group | 12 | PHQ-9 | Active control: e-journal | Decrease in depressive symptoms of both groups | Low -2 |
| Limmroth et al ^28^ | Prospective and retrospective noninterventional observational cohort study | Mobile app | PwMS | Gamified cognitive training | 52 | EQ-5D-5L | n/a | No difference in HRQoL at 6 or 12 months from baseline | High - 4 |
| McArthur et al ^29^ | Mixed methods | Teleconference using Zoom | PwMS | Self-learning and support group for fall prevention | 6 | MS QoL -54 | n/a | No significant change in QoL between visits 1 to 3 | High - 4 |
| Moss-Morris et al ^30^ | Pilot RCT | MSinvigor-8 -Website providing education and information along with reminder emails | PwMS | Educational, CBT, self-management | 10 | HADS | Control | Greater reduction in anxiety and depression in the intervention group compared to control after 10 weeks | Low - 1 |
| Najafi et al ^31^ | Single blind RCT | Tele-conference and social media | PwMS | Tele management of yoga or Pilates | 8 | BDI;  MSQoL-54; GHQ | Control | Improvement in depression and QoL in intervention groups compared to control | Moderate - 3 |
| Pagliari et al ^32^ | Multicentre, rater-blinded, active controlled RCT | Integrated virtual reality | PwMS | Virtual reality cognitive training | 6 | MsQoL-54  BDI | Usual care treatment control | Greater improvement in QoL in intervention than in control. However, both groups reported significant lower depressive symptoms | High - 5 |
| Paul et al ^33^ | RCT | Website | PwMS | Self-management | 12 | LMSQoL; HADS;  MSIS-29 | Usual care | No significant difference in anxiety, QoL and depression in intervention compared to control group. However, anxiety improved in control | High - 4 |
| Pottgen et al ^34^ | Three-armed controlled multi-centre trial with two intervention groups (MaTiMS and MaTiMS plus BrainStim) | Computer software | PwMS | Educational; neuropsychological exercises and treatment | 3 | HADS; HAQUAMS | Standard intervention | Group showed improvements in anxiety, depression and QoL in intervention groups but not sustained at 6 months. | Low - 2 |
| Pratap et al ^35^ | Prospective health population comparison | ElevateMS - Mobile app | PwMS  People without MS | Daily self-functional assessments | 12 | Neuro-QoL | No MS Controls | No change in QoL in relation to intervention functional tests except scores from severe category of instrument. | High - 5 |
| Sadeghi et al ^36^ | Single centre prospective RCT | Tele-consultation | PwMS; | Tele-management of general health status | 52 | BDI;  HADS;  MSIS-29 | Standard care | No significant change in depression, anxiety or HRQoL in the intervention group | High - 4 |
| Saladino et al ^37^ | Prospective cohort multicentre study | Virtual reality computer software | PwMS | Neurorehabilitation and telerehabilitation | 24 | BDI;  MusiQoL | n/a | Intervention resulted in significant positive difference in depression and QoL | High - 5 |
| Sangelaji et al ^38^ | Mixed-methods case study | Website | PwMS | Web physiotherapy services; diary for progress monitoring | 24 | MSIS - 29 V2 HADS | n/a | 50% of participants reported higher QoL following intervention | Low - 1 |
| Sesel et al ^39^ | RCT | Website | PwMS | Mindfulness-based intervention for chronic intervention | 8 | CES-D: Center for Epidemiological Studies–Depression Scale;  GAD-7;  MSIS-29 | Wait-list control | Intervention group reported decreased depression and HRQoL unrelated to depression history. No difference in anxiety reported in intervention group | Moderate -3 |
| Tarakci et al ^40^ | Prospective single-blind RCT | Video phone calls | PwMS | Telerehabilitation exercise program | 12 | Qol Scale (QoLS);  NHP | Physical therapy Rehabilitation | No significant between-group difference in QoL | High -4 |
| Turkowitch et at ^41^ | Clinical trial | Teleconference - Zoom | PwMS | Tele-CBT | 6 | PHQ-9; GAD-7; MSIS-29 | Face-to-face CBT | Intervention group showed no significant difference in depression, anxiety or QoL compared to control at 6 weeks | High - 4 |
| Turner et al ^42^ | Two-group single-blind RCT | Telehealth; home monitoring unit using web-based graphical interface or cell phones | PwMS | Telehealth monitoring to promote physical activity; Provision of tele-counselling | 24 | Depression Module of the PHQ-9 | Self- directed education | Lower depression reported in intervention compared to control group | High - 5 |
| Van Beek et al ^43^ | Proof of concept RCT | Tablet app | PwMS | Self-management; tele-exercises also using virtual reality | 4 | MSIS-29 | Theraband intervention | Significant improvement in HRQoL within intervention group compared to control group but not sustained at 12 weeks. | High -5 |
| Van Geel et al ^44^ | Pre-post intervention | App | PwMS | Self-management walking activities; virtual coaching; support group | 10 | SF-36;  MSIS-29 | n/a | No significant change in HRQoL following intervention and at 10 weeks | High - 4 |
| Van Kessel et al ^45^ | Pilot RCT | Website | PwMS | Information sharing; self-management | 10 | HADS | MSInvigor8-Only | No between-group difference in anxiety or depression even over 10 weeks | Moderate - 3 |
| Wingo et al ^46^ | Prospective single-group pilot study | Mobile app/ website; App generated push text; emails | PwMS | Education; Diary Journal for progress tracking; tele-coaching | 12 | PHQ-9;  RAND 36-Item Health Survey; GAD-7 | n/a | Only mood showed significant improvement following intervention | High - 4 |
| Zissman et al ^47^ | Randomised prospective study | Video-conference | PwMS | Information support | 24 | MSQoL54 | Standard care | Improvement in HRQoL in both groups but to a more significant degree in the intervention compared to control group | High - 4 |

*BDI - Beck depression inventory- II*

*CBT – Cognitive behavioural therapy*

*CES-D – Centre for Epidemiological Studies Depression Scale*

*EQ-5D-5L - EuroQol quality of life measure*

*FAMS – Functional Assessment of Multiple Sclerosis*

*GAD – 7 - Generalized Anxiety Disorder 7-item Scale*

*GHQ -12 - General Health Questionnaire*

*HADS - Hospital Anxiety and Depression Scale*

*HAQUAMS - Hamburg Quality of Life Questionnaire in Multiple Sclerosis*

*HRQoL – Health related quality of life*

*KHQ - Kings Health Questionnaire*

*LMSQoL - Leeds Multiple Sclerosis Quality of Life*

*MOOC – Massive open online course*

*MS – Multiple sclerosis*

*MSIS-29 - Multiple Sclerosis Impact Scale*

*MSQoL-54 – Multiple Sclerosis Quality of Life -54*

*MusiQoL- Multiple Sclerosis International Quality of Life questionnaire*

*Neuro-QoL - Quality of Life in Neurological Disorders*

*NHP – Nottingham Health Profile*

*PHQ-9 - Patient Health Questionnaire*

*PwMS – People with multiple sclerosis*

*QoL – Quality of life*

*QoLS- Quality of life scale*

*RCT – Randomised controlled trial*

*SF36 – Short Form 3654*

*STAI - State-Trait Anxiety Scale*

*WHO-QOL BREF- WHO Quality of Life Scale*

**Supplementary material 4: Demography of included articles**

| **Author and year** | **Intervention characteristics** | **Participant numbers** | **Total number of participants** | **Participant gender (female %)6** | **Participant age mean/ range (SD)** | **Participant MS Type – number (%)** | **EDSS Score (SD)** | **Years with MS -number (SD)** | **Participant occupational status** | **Control/ comparison group ( Number)** | **Control Ms Type (if applicable/ MS) - numbers (%)** |
| --- | --- | --- | --- | --- | --- | --- | --- | --- | --- | --- | --- |
| Alschuler et al ^1^ | Psychoeducation and skills with positive psychology training | 31 | PwMS – (15) | 83.30% | 59.8 (7.7) | RRMS 83.3% SPMS 16.7% | Nr | 18.6 (16.3) | Nr | Wait-list control – (16) | RRMS 56.3% SPMS 18.8% PPMS 25.0% |
| Bessing et al ^2^ | Course providing knowledge and information about MS | 560 | PwMS – (213) No Ms – (347) | 80.75% | PwMS: 50.68 (10.98)  No MS: 52.23 (12.92) | Nr | Nr | 9.33 (8.57) | Nr | n/a | n/a |
| Boeschoten et al ^3^ | Psychoeducation; self-management support | 44 | PwMS – (44) | 77% | 45 (12) | RRMS 48%  SPMS 23% PPMS 18 % Unknown 11% | Nr | 5 (2–40)* [onset] | Nr | n/a | n/a |
| Bogosian et al ^4^ | Mindfulness based cognitive therapy | 40 | PwMS – (19) | 47.40% | 53.42 (8.3) | PPMS 42%  SPMS 57.5% | 6.8 (1.6) | 16.24 (10.1) | Nr | Wait list control –(21) | PPMS 12 (57.1%)  SPMS 9 |
| Bulbul et al ^5^ | Telerehabilitation of pelvic floor muscle training; feedback support | 50 | PwMS – (25) | 100% | 36.76 (9.32) | Nr | <6.5 | 9 (2-26) | Nr | Life-style only advice – (25) | N/a |
| Cavalera et al ^6^ | Group psychoeducation course | 139 | PwMS – (69) | 67% | 42.26 (8.35) | RRMS 51 (94%) SPMS 3 (6%) | 3 (median) | 11.19 (8.0) | Nr | Active control : Online psychoeducation – (70) | RRMS 62 (92%)  SPMS 5 (8%) |
| Chen et al ^7^ | Symptom monitoring | 45 | PwMS – (45) | 91.11% | 41.69 (13.39) | RRMS 39 (86.67%)  PPMS 3 (6.67%) SPMS 2 (4.44%)  Unsure 1 (2.22%) | Score 0-2 – 5 (11.11)  Score 2.5 - 12 (26.67)  Score 3 - 14 (31.11)  Score 3.5 - 4.5 11 (24.45)  Score >4.5 – 3 (6.66) | 11.06 (9.30) | Nr | n/a | n/a |
| Chikersal et al ^8^ | Remote monitoring | 56 | PwMS – (56) | 86% | 43.5 (37-52) | Nr | Nr | Nr | Nr | n/a | n/a |
| Claflin et al ^9^ | Education; information support | 813 | PwMS – (813) | 80.60% | 47.0 (11.9) | Nr | Nr | 8.5 (8.5) | Nr | n/a | n/a |
| Claflin et al ^10^ | Education | 357 | PwMS (213)**  caregiver (144) | 85.50% | 51.6 (12.2) | Nr | Nr | 8.7 (8.5) | Nr | n/a | n/a |
| Dogru-Huzmeli et al ^11^ | Telerehabilitation - Cawthorne Cooksey exercises | 1 | PwMS- (1) | 0% | 39 | RRMS | 1 | 15 | Nr | n/a | n/a |
| Donkers et al ^12^ | Telephysiotherapy exercises | 48 | PwMS – (32) | 63% | 54.6 (11.9) | Nr | Nr | 20.0 (11.3) | Nr | Active comparator: usual care exercise – (16) | N/a |
| Dunne et al ^13^ | Online mindfulness programs | 55 | PwMS: 1) Mindfulness for Multiple Sclerosis (M4MS) (18); 2) Chair Yoga (18) | 82.40% | M4MS: 44.6 (10.1)  Yoga: 48.2 (10.4) | Nr | Nr | M4MS 8 (10.0) Yoga 7.5 (12.0) IQR | Nr | Wait-list control – (19) | N/a |
| Fischer et al ^14^ | Tele-CBT; education | 90 | PwMS –(45) | 76% | 45·36 (12·64) | Clinically isolated syndrome 3 (7%) RRMS 21 (47%) SPMS 9 (20%) PPMS 7 (16%) Unclear 5 (11%) | Nr | 8·20 (7·31) | Nr | Waitlist control – (45) | Clinically isolated syndrome 3 (7%) RRMS 19 (42%) SPMS 12 (27%)  PPMS 4 (9%) Unclear 7 (16% |
| Flachenecker et al ^15^ | Tele-exercise and physical activity; Diary progress tracking | 84 | PwMS – (42) | 65% | 47.6 (9.2) | RRMS 9 (56%) | 4.3 median | 13.4 (7.9) | Nr | Control – (42) | RRMS 20 (67%) |
| Gandy et al ^16^ | Online psycho-education - CBT; self-management | 85 | PwMS (41), (separate analysis) | Nr | >18^ | Nr | Nr | Nr | Nr | Waitlist Control – (44) | n/a |
| Gandy et al ^17^ | Tele-CBT; education and cognitive rehabilitation | 29 | PwMS – (29) | Nr | >18^ | Nr | Nr | Nr | Nr | n/a | n/a |
| Golan et al ^18^ | Diary for symptom monitoring; monthly reporting | 97 | PwMS – (97) | 66% | 40.4 (11) | RRMS 90 (93%) SPMS 6 (6%) PPMS 1 (1%) | 3.4 (2.1) | 9.1 (8.0) | Nr | n/a | n/a |
| Halstead et al ^19^ | Psychoeducation and practical skills | 62 | PwMS (31); Caregivers (31) dyads ( spouses/ children, parent ) | 80.60% | 18 - 65 | RRMS 29  SPMS 1  PPMS 1 | Nr | 13.2 | Employed full time 5 Employed part time 1 Unemployed 20 Other 5 | n/a | n/a |
| Jeong et al ^20^ | Exercise telerehabilitation; self-management | 45 | PwMS (Nr) | 79.30% | 57.8 (11.9) | Nr | 5.5 to 7.5 | Nr | Nr | MS bulletin control group (Nr) | N/a |
| Jongen et al ^21^ | Self-management support | 105 | PwMS (105) | 82.80% | 44.2 (10.6) | Nr | Nr | Nr | Nr | n/a | N/a |
| Kahraman et al ^22^ | Telerehabilitation-based motor imaging training (Tele-MIT) | 70 | PwMS – (25)  No MS | 80% | 34.5 | Nr | 1 | Nr | Employee 11 (55%) Non-employee 5 (25%) Retired 2 (10%) Student 2 (10%) | Control – (25),  No MS control – (20) | N/a |
| Kever et al ^23^ | Support group | 31 | PwMS (20) | 90% | 39.5 (median) | Nr | Nr | 7 (1.5) | Nr | No treatment control –(11) | N/a |
| Khazaeili et al ^24^ | Remote mindfulness-based cognitive therapy and stress therapy; social media support group | 30 | caregivers (15) (RRMS pts) | 100% | 20 -70 | Nr | Nr | Nr | Nr | No intervention control – (15) | N/a |
| Kratz et al ^25^ | Symptom self-management; Tele-CBT | 20 | PwMS (20) | 65% | 48.05 (12.16) | RRMS 10 (50%) PPMS 3 (15%) SPMS 4 (20%) Unknown MS subtype 3 (15%) | Nr | 9.65 (7.48) | Nr | n/a | n/a |
| Landtblom et al ^26^ | Remote monitoring; information support; diary for progress monitoring | 93 | PwMS: [My Support plus (MSP)] – (46) | 63% | MSP - 41 (13.2) | RRMS | **MSP:** Score >4 ~ 11%;  Score < or = 4 ~ 89% | Nr | Nr | Technical support – (47) | RRMS |
| Leavitt et al ^27^ | Support group | 24 | PwMS (14) | 64.30% | 43.0 (10.8) | Nr | Nr | 8.6 (5.4) | Nr | Active control: e-journal - (10) | N/a |
| Limmroth et al ^28^ | Gamified cognitive training | 62 | PwMS (62) | 66% | 43.2 (11.5) | Nr | Nr | Nr | Nr | n/a | n/a |
| McArthur et al ^29^ | Self-learning and support group for fall prevention | 18 | PwMS (18) | 89% | 64.5 (10.71) | Nr | Nr | 29.83 (13.16) | Nr | n/a | n/a |
| Moss-Morris et al ^30^ | Educational, CBT, self-management | 45 | PwMS (23) | 69.60% | 40.14 (17.76) | RRMS 43.5% SPMS 30.4% PPMS 8.7% Unsure 17.4% | Nr | 21 (9.05) | Working less - 9% Unemployed - 31.8% | Control group – (22) | RRMS 70.6% SPMS 11.8% Unsure 17.6% |
| Najafi et al ^31^ | Tele management of yoga or Pilates | 45 | PwMS  [Tele-Pilates – (15)  Tele-Yoga (15)] | 100% | Tele-pilates: 36.2 (4.33)  Tele-yoga: 37.4 (6.03) | RRMS | Tele-pilates 2.5 (1.32) Tele-yoga 2.5 (1.19) | Tele-pilates 10.93 (4.38) Tele-yoga 8.00 (5.84) | Nr | No intervention control (15) | RRMS |
| Pagliari et al ^32^ | Virtual reality cognitive training | 70 | PwMS (35) | 60% | 48.33 (9.66) | Nr | 5.00 | 12.68 (6.72) | Nr | Usual care treatment – (35) | n/a |
| Paul et al ^33^ | Self-management | 30 | PwMS – (15) | 80% | 50.8 (7.4) | Benign 0 PPMS 1 RRMS 11 SPMS 2 Not known 1 | 6.0 (0.5) | 12.5 (7.1) | Nr | Usual care – (15) | Benign 2 PPMS 3 RRMS 6 SPMS 3 Not known 1 |
| Pottgen et al ^34^ | Educational; neuropsychological exercises and treatment | 288 | PwMS Intervention 1: (108)  Intervention 2 : (68) | 69% | **IG1**: 42.77 (10.23) **IG2**: 43.24 (9.29) | **IG1**  RRMS 77(71%) SPMS 15(14%) PPMS 16(15%)  **IG2**  RRMS 59 (87%) SPMS 7 (10%) PPMS 2 (3%) | **IG1:** 3.42 **IG2:** 2.77 | **IG1**: 8.90 (7.52) **IG2:** 9.06 (7.90) | Nr | Standard intervention – (112) | RRMS 81 (72) SPMS 17 (15) PPMS 14 (13) |
| Pratap et al ^35^ | Daily self-functional assessments | 660 | PwMS- (495)**  No MS | 73.30% | **Self-reported** -45.20 (11.64)  **Referred** 48.93 (11.20) | **Self-reported MS**  RRMS 83.6% PPMS 9.5% SPMS 7%  **Neurologist referred**  RRMS 90.4% PPMS 4.4% SPMS 3.7%  Not sure 1.5% | Nr | **Self-reported** 11.14 (8.86)  **Neurologist referred**  14.29 (8.89) | **Self-reported**  Full time 43.5% Part-time 7.9% Retired 8.4% Disabled 29% Unemployed 4.7%  Other 6.5%    **Neurologist referred:**  Full time 44.6% Parttime 12.0% Retired 10.9% Disabled 20.7% Unemployed 3.3%  Other 8.7% | No MS- (134) | N/a |
| Sadeghi et al ^36^ | Tele-management of general health status | 60 | PwMS (30) | 63.30% | 41.3 (10.4) | RRMS 21  SPMS 8  PPMS 1 | 4.2 (2.1) | 10.1 (7.1) | Unemployed 1 Employed (active) 14  sick leave /(temporary) 0 disability leave (permanent) 14 Retired 1 | Standard care - 30 | RRMS 21 SPMS 7 PPMS 2 |
| Saladino et al ^37^ | Neurorehabilitation and telerehabilitation | 68 | PwMS  [Institutional VR **(IVR**) (54)  Tele-rehabilitation **(TR**) (14)] | 57.40% | **IVR**: 44.7 (18–77)    **TR**: 41.7 (25–58) | **VR**  PPMS 4  SPMS 8 RRMS 42  **TR**  PPMS 1  SPMS 0  RRMS 13 | **IVR** - 4.0 (median) | Nr | **VR**:  Full time 18 Part time job 9 License* 4 Retired 5 Retired due to disability 10 Unemployed 8  **TR**:  Full time 7 Part time 1 License 0 Retired 1 Retired due to disability 3 Unemployed 2 | n/a | n/a |
| Sangelaji et al ^38^ | Web physiotherapy service; diary for progress monitoring | 4 | PwMS – (4) | 100% | **76; 65; 56; 56** | **Unknown 1 SPMS 2**  **RRMS 1** | **Nr** | Since: 1995 1982 2014 1986 | Nr | n/a | n/a |
| Sesel et al ^39^ | Mindfulness-based intervention for chronic intervention | 132 | PwMS (69) | Nr | 45.13 (10.74) | RRMS 60 (86.96%)  PPMS 3 (4.35%) SPMS 2 (2.9%) Don’t know 4 (5.8%) | 2.45 (1.97) | 9.64 (8.3) | Full-time 21 (30.43)  Part-time 27 (20.45)  Full-time student 3 (4.35) Unemployed/seeking employment 6 (8.7) Registered disability 5 (7.25) Retired 7 (10.14) | Wait-list control – (63) | RRMS 53 (84.13)  PPMS 3 (4.76)  SPMS 3 (4.76)  Don’t know 4 (6.35) |
| Tarakci et al ^40^ | Telerehabilitation exercise program | 41 | PwMS – (20) | 73% | 39.46 (10.59) | RRMS | 3.46 (1.31) | 8.86 (4.50) | Nr | Rehabilitation with physical therapist – (21) | RRMS |
| Turkowitch et at ^41^ | Tele-CBT | 21 | PwMS (11) | 81.80% | 50.3 (13.5) | RRMS | Nr | Nr | Working 4 Retired 3 Unemployed 0 Other 4 | Face to face CBT I – (10) | RRMS - 8 SPMS - 2 |
| Turner et al ^42^ | Telehealth monitoring to promote physical activity; Provision of tele-counselling | 64 | PwMS (31) | 29% | 52.7 (11.6) | RRMS 65.5% Others 34.5% | Nr | Nr | Nr | Self- directed education (EC) (33) | RRMS 69.7% Other 30.3% |
| Van Beek et al ^43^ | Self-management; tele-exercises also using virtual reality | 48 | PwMS: TAD-MS (26) | 81% | 50.84 (14.84) | RRMS 17 (65%) PPMS 6 (23%) SPMS 3 (12%) | 2.98 (1.81) | 12.33 (8.23) | Nr | Theraband intervention – (22) | RRMS15 (68%)  PPMS 5 (23%) SPMS 2 (9%) |
| Van Geel et al ^44^ | Self-management walking activities; virtual coaching; support group | 12 | PwMS (12) | 100% | 42.5 | RRMS 11  SPMS 1 | - | 7 years (IQR = 4.5–11) | Nr | n/a | n/a |
| Van Kessel et al ^45^ | Information sharing; self-management | 39 | PwMS MSInvigor8-Plus– (19) | 58% | 42.95 (8.16) | RRMS 15 (79.0%)  SPMS 2 (10.5%) Not known 2 (10.5%) | Score 0–4 53.0%  Score 4.5–5.5 10.5%  Score 6–6.5 31.5%  Not recorded 5.0% | 4.78 (4.36) | Working 11 (58.0)  Unemployed/retired 5 (26.0) Student 2 (10.5) Housewife/husband 1 (5.5) | MSInvigor8-Only – (20) | RRMS 11 (55.0%)  SPMS 3 (15.0%)  Not known 6 (30.0%) |
| Wingo et al ^46^ | Education; Diary Journal for progress tracking; tele-coaching | 20 | PwMS (20) | 85% | 46.15 (11.60) | RRMS | 3.25 | Nr | Nr | n/a | n/a |
| Zissman et al ^47^ | Information support | 40 | PwMS (20) | 85% | 43.8 (11.5) | RRMS | Nr | 7.9 (6.6) | Nr | Standard care – (20) | RRMS |

CBT – Cognitive behavioural therapy

M4MS - Mindfulness for Multiple Sclerosis

MSP - MySupportPlus

N/a - not applicable

No MS - No multiple sclerosis

Nr - Not reported

TAD-MS - Tablet app-based dexterity training in multiple sclerosis

*disease onset

** demography only provided for completers

**Supplementary material 5:** List of Excluded Articles from Full-Text Screening

1. Abbadessa G, Lavorgna L, Miele G, et al. (2021) Assessment of multiple sclerosis disability progression using a wearable biosensor: A pilot study. *JOURNAL OF CLINICAL MEDICINE* 10(6): 1-8.
2. Alberts JL (2017) Utilizing mobile technology in the assessment and monitoring of MS patients. *MULTIPLE SCLEROSIS JOURNAL* 23(3): 18-19.
3. Altmann P, Ponleitner M, Leutmezer F, et al. (2022a) haMSter: a Smartphone Application for Remote Patient Monitoring. *Neurology* 98(18).
4. Altmann P, Ponleitner M, Leutmezer F, et al. (2021) haMSter: A smartphone application for remote patient monitoring in multiple sclerosis. *MULTIPLE SCLEROSIS JOURNAL* 27(2): 298-299.
5. Altmann P, Ponleitner M, Monschein T, et al. (2022b) Feasibility of a smartphone app to monitor patient reported outcomes in multiple sclerosis: The haMSter interventional trial. *DIGITAL HEALTH* 8.
6. Baetge SJ, Filser M, Renner A, et al. Supporting brain health in multiple sclerosis: exploring the potential of neuroeducation combined with practical mindfulness exercises in the management of neuropsychological symptoms. *JOURNAL OF NEUROLOGY*. DOI: doi:10.1007/s00415-023-11616-2.
7. Balasubramanian GV, Beaney P and Chambers R (2021) Digital personal assistants are smart ways for assistive technology to aid the health and wellbeing of patients and carers. *BMC Geriatrics* 21(1).
8. Basirat A, Raeisi Shahraki H, Farpour HR, et al. (2020) The Correlation between Using Social Networks and the General Health of Multiple Sclerosis Patients. *MULTIPLE SCLEROSIS INTERNATIONAL* 2020.
9. Beadnall HN, Wang C, Benedict RH, et al. (2015) Tablet-based waiting room cognitive screening in a multiple sclerosis clinic. *Multiple Sclerosis* 23(11): 124-125.
10. Becker H, Stuifbergen A, Britt K, et al. (2022) How People with a Chronic Health Condition Have Promoted their Health during COVID-19. *Archives of Physical Medicine & Rehabilitation* 103(3): e40-e41.
11. Bosa C, Schillaci V, Alteno A, et al. (2021) Impact of COVID-19 lockdown on a population of progressive multiple sclerosis patients in Northern Italy. *MULTIPLE SCLEROSIS JOURNAL* 27(2): 205-206.
12. Bove R, Vaughan T, Healy BC, et al. (2012) Patients can adequately report their ms severity online; implications for online research platforms. *Annals of Neurology* 72: S111.
13. Bove RM, Rush G, Zhao C, et al. (2019) A Videogame-Based Digital Therapeutic to Improve Processing Speed in People with Multiple Sclerosis: A Feasibility Study. *NEUROLOGY AND THERAPY* 8(1): 135-145.
14. Broadbent FJ and Swalwell JM (2020) "I can do more than I thought I could": exploring the online blogs from the Sailing Sclerosis Oceans of Hope journey. *DISABILITY AND REHABILITATION* 42(6): 880-886.
15. Caceres FJ, Saladino ML, Scaffa ME, et al. (2019) Neuro rehabilitation effectiveness based on virtual reality and tele rehabilitation in patients with multiple sclerosis in Argentina. 'Reavitelem' study. *MULTIPLE SCLEROSIS JOURNAL* 25: 352-353.
16. Castejón N, Chekroun M, Martínez García J, et al. (2013) Patient networks as a data source for patient reported outcomes research. carenity experience. *VALUE IN HEALTH* 16(7): A608.
17. Chan W, Dobbs B, Shaw M, et al. (2017) Baseline affect predicts improved fatigue with telerehabilitation using remotely-supervised transcranial direct current stimulation (RS-tDCS) in adults with multiple sclerosis (MS). *Neurology* 88(16).
18. Charron O, Onuorah H, Montague A, et al. (2021) Impact of the COVID-19 pandemic on healthcare access and perceived outcomes: A survey study of people with MS in the United States. *MULTIPLE SCLEROSIS JOURNAL* 27(2): 686-687.
19. Chen MH, Goverover Y, Botticello A, et al. (2022a) Healthcare Disruptions and Use of Telehealth Services Among People With Multiple Sclerosis During the COVID-19 Pandemic. *Arch Phys Med Rehabil* 103(7): 1379-1386.
20. Chen MH, Leow A, Ross MK, et al. (2022b) Associations between smartphone keystroke dynamics and cognition in MS. *DIGITAL HEALTH* 8.
21. Cheng WY, Bourke AK, Lipsmeier F, et al. (2021) U-turn speed is a valid and reliable smartphone-based measure of multiple sclerosis-related gait and balance impairment. *Gait and Posture* 84: 120-126.
22. Claflin S, Campbell J, Bessing B, et al. (2022) Knowledge translation in the international MS community: an evaluation of the Understanding Multiple Sclerosis free online course. *MULTIPLE SCLEROSIS JOURNAL* 28(4): 19-20.
23. Clinch S, McDougall F, Barrett A, et al. (2021) A patient-focused qualitative study to support content validity of digital performance assessments in MS. *MULTIPLE SCLEROSIS JOURNAL* 27(2): 329.
24. Cogley C, Davenport L, Monaghan R, et al. (2021) Evaluating the effectiveness and acceptability of an ACT informed neuropsychology group for individuals with MS, delivered using video conferencing. *MULTIPLE SCLEROSIS JOURNAL* 27(2): 191-192.
25. Craven MP, Andrews JA, Lang AR, et al. (2020) Informing the Development of a Digital Health Platform Through Universal Points of Care: Qualitative Survey Study. *JMIR Form Res* 4(11): e22756.
26. D'Hooghe M, Van Gassen G, Kos D, et al. (2015) Evaluating the effect of enhanced physical activity and energy management on fatigue in patients suffering from multiple sclerosis: The MS TeleCoach study. *Multiple Sclerosis* 23(11): 611-612.
27. Dennison L, Brown M, Kirby S, et al. (2016) Prognosis in multiple sclerosis: A UK national survey. *Multiple Sclerosis* 22: 109.
28. Dorstyn D, Roberts R, Murphy G, et al. (2018) Online Resource to Promote Vocational Interests Among Job Seekers With Multiple Sclerosis: A Randomized Controlled Trial in Australia. *ARCHIVES OF PHYSICAL MEDICINE AND REHABILITATION* 99(2): 272-280.
29. Dorstyn D, Roberts R, Murphy G, et al. (2015) Online vocational rehabilitation for job-seekers with MS: Does it work? *Multiple Sclerosis* 21(14): 20-21.
30. Ehde DM, Arewasikporn A, Nelson IK, et al. (2017) A pilot randomized controlled trial evaluating the effects of a resilience intervention on adults aging with multiple sclerosis. *MULTIPLE SCLEROSIS JOURNAL* 23(3): 671-672.
31. Emmert A, Rodgers JD, Kininger R, et al. (2013) Cognitive dysfunction predicts negative work events and accommodations in multiple sclerosis. *Multiple Sclerosis* 19(7): 981.
32. Engelhard MM, Patek SD, Sheridan K, et al. (2017) Remotely engaged: Lessons from remote monitoring in multiple sclerosis. *International Journal of Medical Informatics* 100: 26-31.
33. Farpour HR, Hoveidaei AH, Habibi L, et al. (2020) The impact of social media use on depression in multiple sclerosis patients. *ACTA NEUROLOGICA BELGICA* 120(6): 1405-1409.
34. Fern, ez-Vazquez D, Cano-de-la-Cuerda R, et al. (2021) Wearable Robotic Gait Training in Persons with Multiple Sclerosis: A Satisfaction Study. *SENSORS* 21(14).
35. Fink K, Kläppe U and Lindblom S (2016) A randomized, multi-center, open-label, observational study to investigate quality of life improvement in patients with relapse remitting multiple sclerosis on first-line treatment that are using a wearable device with biofeedback for stress. *Multiple Sclerosis* 22: 819-820.
36. Fischer A, Schröder J, Pöttgen J, et al. (2013) Effectiveness of an internet-based treatment programme for depression in multiple sclerosis: A randomized controlled trial. *Multiple Sclerosis* 19(11): 350-351.
37. Fitzgerald KC, Salte A, Tyry T, et al. (2019) Validation of the SymptoMScreen with performance-based or clinician-assessed outcomes. *MULTIPLE SCLEROSIS AND RELATED DISORDERS* 29: 86-93.
38. Fjeldstad C, Thiessen A and Pardo G (2016) Telerehabilitation in multiple sclerosis: Results of a randomized, 3-arm, rater blinded, feasibility and efficacy pilot study; patient-reported outcomes report. *Multiple Sclerosis* 22: 390.
39. Frontario A, Feld E, Sherman K, et al. (2016) Telehealth mindfulness meditation improves cognitive performance in adults with multiple sclerosis (MS). *Neurology* 86(16).
40. Fuchs T, Ziccardi S, Benedict R, et al. (2018) Disease course and grey matter volume predict success of home-based cognitive rehabilitation in multiple sclerosis. *MULTIPLE SCLEROSIS JOURNAL* 24(2): 518-519.
41. Giunti G, Kool J, Romero OR, et al. (2018) Exploring the Specific Needs of Persons with Multiple Sclerosis for mHealth Solutions for Physical Activity: Mixed-Methods Study. *JMIR mHealth and uHealth* 6(2).
42. Golan D, Sagiv S, Glass-Marmor L, et al. (2021) Mobile-phone-based e-diary derived patient reported outcomes: Association with clinical disease activity, psychological status and quality of life of patients with multiple sclerosis. *PLoS ONE* 16(5): e0250647.
43. Golan D, Sagiv S, Ratzabi S, et al. (2017) The use of a mobile-phone-based E-diary for evaluation of patient-reported outcomes and adherence to treatment of patients with multiple sclerosis. *MULTIPLE SCLEROSIS JOURNAL* 23(3): 739.
44. Gold SM, Friede T, Meyer B, et al. (2022) Online intervention to reduce depressive symptoms in multiple sclerosis: an international multicenter randomized controlled phase III trial. *MULTIPLE SCLEROSIS JOURNAL* 28(3): 89-90.
45. Goodwin RA, Lincoln NB, das Nair R, et al. (2020) Evaluation of NeuroPage as a memory aid for people with multiple sclerosis: A randomised controlled trial. *NEUROPSYCHOLOGICAL REHABILITATION* 30(1): 15-31.
46. Greiner P, Sawka A and Imison E (2015) Patient and Physician Perspectives on MSdialog, an Electronic PRO Diary in Multiple Sclerosis. *PATIENT-PATIENT CENTERED OUTCOMES RESEARCH* 8(6): 541-550.
47. Griffin N and Kehoe M (2018) A questionnaire study to explore the views of people with multiple sclerosis of using smartphone technology for health care purposes. *DISABILITY AND REHABILITATION* 40(12): 1434-1442.
48. Grothe L, Speerfock S, Schomerus G, et al. (2022) Social media use and its impact on stigma, coping, and quality of life in patients with multiple sclerosis. *MULTIPLE SCLEROSIS JOURNAL* 28(3): 732.
49. Guijarro-Castro C, Aladro-Benito Y, Sánchez-Musulim A, et al. (2017) Face-to-Face or Telematic Cognitive Stimulation in Patients with Multiple Sclerosis and Cognitive Impairment: Why Not Both? *Behavioural Neurology* 2017.
50. Guo C, Cahir-Mcfarl, E, et al. (2020) Konectomtm smartphone-based digital outcome assessment of cognitive and motor function in multiple sclerosis. *MULTIPLE SCLEROSIS JOURNAL* 26(3): 168.
51. Guo G, Zhang HB, Yao LY, et al. (2021) MSLife - Digital Behavioral Phenotyping of Multiple Sclerosis Symptoms in the Wild Using Wearables and Graph-Based Statistical Analysis. *PROCEEDINGS OF THE ACM ON INTERACTIVE MOBILE WEARABLE AND UBIQUITOUS TECHNOLOGIES-IMWUT* 5(4).
52. Hadgkiss EJ, Jelinek GA, Taylor KL, et al. (2015) Engagement in a program promoting lifestyle modification is associated with better patient-reported outcomes for people with MS. *Neurol Sci* 36(6): 845-852.
53. Harrison AM, McCracken LM, Jones K, et al. (2017) Using mixed methods case-series evaluation in the development of a guided self-management hybrid CBT and ACT intervention for multiple sclerosis pain. *Disability & Rehabilitation* 39(18): 1785-1798.
54. Heine M, Beckerman H, Hämäläinen P, et al. (2020) Evidence-Based Rehabilitation for Multiple Sclerosis Made Easy: The Online Applying Evidence with Confidence (APPECO) Platform. *International Journal of MS Care* 22(6): 263-269.
55. Houlihan BV, Jette A, Friedman RH, et al. (2013) A pilot study of a telehealth intervention for persons with spinal cord dysfunction. *Spinal Cord* 51(9): 715-720.
56. Howard IM and Burgess K (2021) Telehealth for Amyotrophic Lateral Sclerosis and Multiple Sclerosis. *PHYSICAL MEDICINE AND REHABILITATION CLINICS OF NORTH AMERICA* 32(2): 239-251.
57. Ja J, Kalincik T, Zhu C, et al. (2022) MSReactor is an acceptable long-term web-based cognitive monitoring platform for patients with multiple sclerosis. *MULTIPLE SCLEROSIS JOURNAL* 28(3): 468-469.
58. Jeong IC, Liu J and Finkelstein J (2020) Association Between System Usage Pattern and Impact of Web-Based Telerehabilitation in Patients with Multiple Sclerosis. *Studies in health technology and informatics* 272: 346-349.
59. Jongen P, Heerings M, Kool A, et al. (2012) Www.msregistry.eu: Interactive online registry for self-monitoring by people with multiple sclerosis and clinically isolated syndrome. *Multiple Sclerosis* 18(4): 320.
60. Jongen P, Sinnige O, Van Geel B, et al. (2013a) MSmonitor: An interactive web-based tool for selfmanagement and long-term treatment monitoring in multiple sclerosis. *Multiple Sclerosis* 19(11): 483-484.
61. Jongen PJ, ers E, Zwanikken C, et al. (2013b) Adherence to monthly online self-assessments for short-term monitoring: A 1-year study in relapsing-remitting multiple sclerosis patients after start of disease modifying treatment. *PATIENT PREFERENCE AND ADHERENCE* 7: 293-300.
62. Jongen PJ, Ter Veen G, Lemmens W, et al. (2020) The Interactive Web-Based Program MSmonitor for Self-Management and Multidisciplinary Care in Persons With Multiple Sclerosis: Quasi-Experimental Study of Short-Term Effects on Patient Empowerment. *J Med Internet Res* 22(3): e14297.
63. Kempcke R, Isermann C, Suhrbier A, et al. (2013) Evaluation of the multiple sclerosis documentation system MSDS. *Multiple Sclerosis* 19(11): 246-247.
64. Kempcke R, Schultheiß T and Ziemssen T (2012) Advances in MS patient management: Update of Multiple Sclerosis Documentation System 'MSDS 3D'. *European Journal of Neurology* 19: 710.
65. Kos D, Raeymaekers J, Van Remoortel A, et al. (2017) Electronic visual analogue scales for pain, fatigue, anxiety and quality of life in people with multiple sclerosis using smartphone and tablet: A reliability and feasibility study. *Clinical Rehabilitation* 31(9): 1215-1225.
66. Kumar D, Bialek A, Divecha A, et al. (2022) Tele-Exercise During COVID-19: Effectiveness of an Adaptive Seated Intervention for Adults With Chronic Neurological Impairments. *ARCHIVES OF PHYSICAL MEDICINE AND REHABILITATION* 103(12): e54-e55.
67. Kunce A, Fritz J and Emery H (2019) Clinical video tele-rehabilitation and neurology followup care in the home for veterans with multiple sclerosis in urban and rural areas. *MULTIPLE SCLEROSIS JOURNAL* 25: 153.
68. Kunce AL and Fritz J (2020) Home delivery of provider care and physical therapy utilizing clinical video telehealth in the va for veterans with MS and ALS. *MULTIPLE SCLEROSIS JOURNAL* 26(1): 142.
69. Lang M, Rau D, Cepek L, et al. (2021) An id-associated application to facilitate patient-tailored management of multiple sclerosis. *BRAIN SCIENCES* 11(8).
70. Latchem-Hastings J, Busse M, Playle R, et al. (2021) LEAP-MS: Co-designing and testing a web-based Lifestyle, Exercise and Activity intervention for People with Progressive Multiple Sclerosis. *MULTIPLE SCLEROSIS JOURNAL* 27(3): 15.
71. Le HV, Kobayashi MG, Kamauu AWC, et al. (2017) Identifying relapsing-remitting multiple sclerosis (RRMS) in united states integrated delivery network healthcare electronic health record data. *Pharmacoepidemiology and Drug Safety* 26: 235-236.
72. Leavitt V, Kever A, Aguerre I, et al. (2020a) The sunlight study: A telehealth intervention to address mental health in persons with MS during COVID-19. *MULTIPLE SCLEROSIS JOURNAL* 26(3 SUPPL): 76-77.
73. Leavitt V, Riley C, De Jager P, et al. (2019) eSupport: A feasibility RCT of internet-based support group participation to reduce loneliness in persons with multiple sclerosis. *MULTIPLE SCLEROSIS JOURNAL* 25: 625-626.
74. Leavitt VM, Riley CS, De Jager P, et al. (2020b) Social network science-based telehealth interventions for MS: Introducing esupport and EFIT. *MULTIPLE SCLEROSIS JOURNAL* 26(1): 47-48.
75. Lerin NM (2021) Subjective impact of online respiratory physiotherapy in people with multiple sclerosis during Covid-19 pandemic. *MULTIPLE SCLEROSIS JOURNAL* 27(3): 11-12.
76. Manacorda T, Battaglia MA, iera P, et al. (2021) Pandemia in the life of persons with MS in Italy. *MULTIPLE SCLEROSIS JOURNAL* 27(2): 733.
77. Marck CH, Hadgkiss EJ, Weil, et al. (2014) Physical activity and associated levels of disability and quality of life in people with multiple sclerosis: a large international survey. *BMC Neurol* 14: 143.
78. Martinez O, Jometon A, Perez M, et al. (2014) Effectiveness of Teleassistance at Improving Quality of Life in People with Neuromuscular Diseases. *SPANISH JOURNAL OF PSYCHOLOGY* 17.
79. Mäurer M (2015) Telerehabilitation in multiple sclerosis. *Multiple Sclerosis* 23(11): 41.
80. Mercier HW, Jette A and Houlihan B (2014) Differential impact and use of a telehealth intervention by persons with multiple sclerosis or spinal cord injury. *ARCHIVES OF PHYSICAL MEDICINE AND REHABILITATION* 95(10): e34-e35.
81. Merlo D, Darby D, Haartsen J, et al. (2018) Cognitive self-monitoring persistence and reliability using the web-based battery, MSReactor. *MULTIPLE SCLEROSIS JOURNAL* 24(2): 354-355.
82. Meshgin D and Kersten-Oertel M (2021) Multiple sclerosis image-guided subcutaneous injections using augmented reality guided imagery. *Computer Methods in Biomechanics and Biomedical Engineering: Imaging and Visualization* 9(4): 370-375.
83. Metzger R, Garrett K, Christensen A, et al. (2022) Digital Performance Measures Show Sensitivity to Demographic and Disease Characteristicsin a Multiple Sclerosis Cohort Utilizing the MS Care Connect Mobile App in a Real-world Setting. *Neurology* 98(18).
84. Miller AE, Cohen BA, Krieger SC, et al. (2014) Constructing an adaptive care model for the management of disease-related symptoms throughout the course of multiple sclerosis - Performance improvement CME. *Multiple Sclerosis* 20(1): 18-23.
85. Monschein T, Leutmezer F and Altmann P (2021) Anwendung von Wearables bei Multipler Sklerose = The use of wearable devices in multiple sclerosis. *Klinische Neurophysiologie* 52(1): 39-43.
86. Moss-Morris R (2012) Cognitive behavioural interventions as a treatment choice for people with MS (#146). *Multiple Sclerosis* 18(5): S6-S7.
87. Müller R, Gertz KJ, Molton IR, et al. (2016) Effects of a tailored positive psychology intervention on well-being and pain in individuals with chronic pain and a physical disability: A feasibility trial. *The Clinical Journal of Pain* 32(1): 32-44.
88. Neal W, Young HJ, Mehta T, et al. (2017) Comparative effectiveness trial between a clinic-and home-based exercise intervention for multiple sclerosis. *ARCHIVES OF PHYSICAL MEDICINE AND REHABILITATION* 98(10): e46.
89. Nørgaard M, Boesen F, Guldhammer Skjerbæk A, et al. (2022) Post-discharge telephone counseling doubles the longterm beneficial effects of inpatient multidisciplinary rehabilitation - The Danish MS Hospitals Rehabilitation Study. *MULTIPLE SCLEROSIS JOURNAL* 28(3): 91.
90. Novotna K and Větrovska R (2021) Use of online pilates program for people with multiple sclerosis during Covid-19 pandemic. *MULTIPLE SCLEROSIS JOURNAL* 27(3): 33-34.
91. Olival G, Santos P, Barbosa G, et al. (2022) Brazilian experience in telehealth for multiple sclerosis during the COVID-19 pandemic. *MULTIPLE SCLEROSIS JOURNAL* 28(2): NP22.
92. Otte C (2015) Online CBT in patients with multiple sclerosis and depression. *The Lancet Psychiatry* 2(3): 192-193.
93. Pardo G, Thiessen A and Fjeldstad C (2016) Telerehabilitation in multiple sclerosis: Results of a randomized, 3-arm, rater blinded, feasibility and efficacy pilot study; gait and balance report. *Multiple Sclerosis* 22: 30.
94. Parks AC, Williams AL, Kackloudis GM, et al. (2020) The effects of a digital well-being intervention on patients with chronic conditions: Observational study. *Journal of Medical Internet Research* 22(1).
95. Pastore F (2021) Nursing digital care during COVID-19 outbreak: The experience of multiple sclerosis centre in southern Italy. *MULTIPLE SCLEROSIS JOURNAL* 27(2): 28-29.
96. Pastore F, Iaffaldano P, Viterbo RG, et al. (2019) SMcare2.0 app: a pilot study to assess the impact of a new nursing e-care application on quality of life of patients with MS. *MULTIPLE SCLEROSIS JOURNAL* 25: 195-195.
97. Paul L, Coulter EH, Miller L, et al. (2013) Physiotherapy-led web-based rehabilitation for people with multiple sclerosis. *Multiple Sclerosis* 19(11): 555-556.
98. Plata-Bello J, Pérez-Martín MY, González-Platas M, et al. (2018) Neuropsychological and brain gray matter volume (GMV) changes after a computerassisted cognitive treatment (CACT) in patients with multiple sclerosis (MS). *European Journal of Neurology* 25: 102.
99. Poettgen J, Moss-Morris R, Wendebourg JM, et al. (2015) Online fatigue management program for patients with multiple sclerosis-a randomized controlled trial. *Multiple Sclerosis* 23(11): 41-42.
100. Prefasi D, Meca-Lallana V, Alabarcez W, et al. (2020) Exploring the usability and patient satisfaction of a virtual rehabilitation program in multiple sclerosis: The rehabvr study protocol. *MULTIPLE SCLEROSIS JOURNAL* 26(3): 157.
101. Pretorius C (2016) The experience of active involvement in an online Facebook support group, as a form of support for individuals who are diagnosed with Multiple Sclerosis. *TYDSKRIF VIR GEESTESWETENSKAPPE* 56(3): 809-828.
102. Pugh S, Patel Y and Morrow T (2017) Feasibility study to evaluate the utility and usage frequency of a digital health coach for multiple sclerosis (MS) patients. *Neurology* 88(16).
103. Rimmer JH, Thirumalai M, Young HJ, et al. (2018) Rationale and design of the tele-exercise and multiple sclerosis (TEAMS) study: A comparative effectiveness trial between a clinic- and home-based telerehabilitation intervention for adults with multiple sclerosis (MS) living in the deep south. *Contemp Clin Trials* 71: 186-193.
104. Robert MK, Hales Reynolds MA, Eisenberg M, et al. (2023) Exploring an 8-Week Online Adaptive Yoga Program for Multiple Sclerosis: A Pilot Study. *Holistic Nursing Practice* 37(2): 62-70.
105. Rudell E, Peterson P, Griffin A, et al. (2020) Using technology to empower patients to manage and track symptoms of MS. *MULTIPLE SCLEROSIS JOURNAL* 26(3): 644.
106. ŞAhin Ş (2020) Management of Neurorehabilitation During the COVID-19 Pandemic. *Duzce Medical Journal* 22: 10-13.
107. Sartori A, Dinoto A, Pasquin F, et al. (2020) Sars-cov-2 pandemic lockdown: Perceived consequences on multiple sclerosis patients. *MULTIPLE SCLEROSIS JOURNAL* 26(3): 105.
108. Schiffmann I, Freund M, Engels K, et al. (2018) Understanding magnetic resonance imaging in multiple sclerosis (UMIMS): Effect of an interactive online education tool on emotions, attitude and knowledge in people with multiple sclerosis. *MULTIPLE SCLEROSIS JOURNAL* 24(2): 522.
109. Sesel AL, Sharpe L, Beadnall HN, et al. (2020) The Evaluation of an Online Mindfulness Program for People with Multiple Sclerosis. *MULTIPLE SCLEROSIS JOURNAL* 26(3): NP14-NP15.
110. Settle JR, Robinson SA, Kane R, et al. (2015) Remote cognitive assessments for patients with multiple sclerosis: A feasibility study. *MULTIPLE SCLEROSIS JOURNAL* 21(8): 1072-1079.
111. Shabalina DO, Zulkaidarova AR, Khramchenko MA, et al. (2022) [Experience of remote rehabilitation for patients with multiple sclerosis]. *Zh Nevrol Psikhiatr Im S S Korsakova* 122(11): 69-73.
112. Sieber C, Chiavi D, Haag C, et al. (2022) Electronic Health Diary Campaigns to Complement Longitudinal Assessments in Persons With Multiple Sclerosis: Nested Observational Study. *JMIR mHealth and uHealth* 10(10).
113. Simblett S, Matcham F, Curtis H, et al. (2020) Patients' Measurement Priorities for Remote Measurement Technologies to Aid Chronic Health Conditions: Qualitative Analysis. *JMIR mHealth and uHealth* 8(6): e15086.
114. Sodre BM, Sim, i TM, et al. (2017) The brief psychology therapy technique in online guidance to patients with multiple sclerosis. *Multiple Sclerosis* 23: 67-68.
115. Sola-Valls N, Blanco Y, Sepulveda M, et al. (2015) Telemedicine for Monitoring MS Activity and Progression. *CURRENT TREATMENT OPTIONS IN NEUROLOGY* 17(11).
116. Solari A, Giordano A, Patti F, et al. (2017) Randomized controlled trial of a home-based palliative approach for people with severe multiple sclerosis. *Multiple Sclerosis* 23(6): 879.
117. Solaro C, Di Giovanni R, Grange E, et al. (2020) Development of an APP (ABOUTCOME) for evaluation in multiple sclerosis. *MULTIPLE SCLEROSIS JOURNAL* 26(2): 32.
118. Sparling A, Stutts L, Sanner H, et al. (2017) In-person and online social participation and emotional health in individuals with multiple sclerosis. *Quality of Life Research* 26(11): 3089-3097.
119. Stahl C, Laub P and Assoc Comp M (2017) Maintaining multiple sclerosis patients' quality of life - a case study on environment control assistance in a smart home. In, pp.83-86. RAYYAN-INCLUSION: {"JOAN"=>"Included", "Adeola"=>"Included"}.
120. Sumner L, Schmidt H, Minden S, et al. (2022) Use of Telemedicine Among People with Multiple Sclerosis Before and During the COVID-19 Pandemic. *Telemed J E Health*. DOI: doi:10.1089/tmj.2022.0284.
121. Tacchino A (2022) Wearable technology in MS. *MULTIPLE SCLEROSIS JOURNAL* 28(3): 60.
122. Tacchino A, D'Amico E, Ponzio M, et al. (2015) Predisposition and motivation assessment in using technologies in multiple sclerosis. A questionnaire on a wearable tool for unobtrusive motor and cognitive monitoring. *Multiple Sclerosis* 21(4): 492.
123. Tallner A, Streber R, Hentschke C, et al. (2016) Internet-supported physical exercise training for persons with multiple sclerosis—a randomised, controlled study. *International Journal of Molecular Sciences* 17(10).
124. tblom AM, Guala D, Hau S, et al. (2017) RebiQoL: A telemedicine patient support program on health related quality of life and adherence in MS patients treated with Rebif. *MULTIPLE SCLEROSIS JOURNAL* 23: 425-425.
125. Teresa T, Margareth Z and Dias A (2022) Online art therapy for multiple sclerosis. *MULTIPLE SCLEROSIS JOURNAL* 28(2): NP22-NP23.
126. Thomas P, Annam H, Ravindu, et al. (2021) Integration of teleneurology within the health system to manage patients of multiple sclerosis and other CNS demyelinating disorders during COVID-19 pandemic. *Annals of Indian Academy of Neurology* 24(3): 443-445.
127. Tietjen KM and Breitenstein S (2017) A Nurse-Led Telehealth Program to Improve Emotional Health in Individuals With Multiple Sclerosis. *JOURNAL OF PSYCHOSOCIAL NURSING AND MENTAL HEALTH SERVICES* 55(3): 31-37.
128. Turner AP and Knowles LM (2020) Behavioral Interventions in Multiple Sclerosis. *Fed Pract* 37: S31-s35.
129. Veldkamp R, Baert I, Kalron A, et al. (2019) Positive effects of a cognitive-motor dual task training compared to a single mobility training on dual task cost are independent of improvements in mobility or cognition. *MULTIPLE SCLEROSIS JOURNAL* 25: 925.
130. Viterbo RG, Pastore F, Guerra T, et al. (2022a) IMPACT OF COVID-19 LOCKDOWN MEASURES ON MENTAL HEALTH IN MULTIPLE SCLEROSIS PATIENTS: THE ROLE OF REMOTE ASSESSMENT. *Neurological Sciences* 43: S514-S515.
131. Viterbo RG, Pastore F, Guerra T, et al. (2022b) Remote assessment of the impact of lockdown measures on mental health in multiple sclerosis patients. *MULTIPLE SCLEROSIS JOURNAL* 28(3): 213-214.
132. Wee SK, Ho CY, Tan SL, et al. (2021) Enhancing quality of life in progressive multiple sclerosis with powered robotic exoskeleton. *MULTIPLE SCLEROSIS JOURNAL* 27(3): 483-487.
133. Weigel M (2021) Evaluation of a 6 week integrative medicine workshop series on quality of life, self-ffficacy and wellness in MS. *MULTIPLE SCLEROSIS JOURNAL* 27(2): 29.
134. Weiss M (2015) Therapeutic software for the treatment of fatigue, depression and anxiety in multiple sclerosis. *Multiple Sclerosis* 23(11): 325.
135. Willis M and Miller D (2017) Tracking quality of multiple sclerosis care: Experience of a large multiple sclerosis center. *Neurology* 88(16).
136. Wilroy JD, Kim Y, Lai B, et al. (2022) How do people with physical/mobility disabilities benefit from a telehealth exercise program? A qualitative analysis. *Front Rehabil Sci* 3: 932470.
137. Wilson-Menzfeld G, Naisby J, Baker K, et al. (2022) Yoga provision for individuals living with Multiple Sclerosis: Is the future online? *PLoS ONE* 17(4).
138. Winkler G and Raji A (2016) Evaluation of e-learning tools for MS patients-mymsmagazin.com. *Multiple Sclerosis* 22: 692.
139. Yavas I, Kahraman T, Sagici O, et al. (2022) Effects of telerehabilitation-based pelvic floor muscle training on urinary incontinence, sexual dysfunction, and quality of life in people with multiple sclerosis: a randomised, controlled, assessor-blinded trial. *MULTIPLE SCLEROSIS JOURNAL* 28(3): 90-91.

**Supplementary material 6:** Distribution of studies deploying CH technologies by outcomes

| **Connected health technology type** | **Anxiety, depression**  **QoL** | **Anxiety**  **depression**  **HRQoL** | **Depression/ Anxiety and QoL/ HRQoL** | **Psychological wellbeing & QoL** | **Anxiety and depression** | **Anxiety depression and other psychological outcome** | **Anxiety alone** | **Depression alone** | **QoL**  **alone** | **HRQoL**  **alone** | **Other psychological outcome** |
| --- | --- | --- | --- | --- | --- | --- | --- | --- | --- | --- | --- |
| Website | 3 studies ^23, 33, 38^ | 1 study ^39^ | 1 study ^14^ |  | 3 studies ^3, 17, 45^ | 2 studies ^12, 19^ |  | 2 studies ^25, 42^ | 2 studies ^2, 9^ | 2 studies ^10, 21^ | 1 study ^27^ |
| Teleconference | 2 studies ^22, 41^ | 1 study ^36^ |  | 1 study ^13^ |  | 2 studies ^1, 4^ |  |  | 4 studies ^5, 11, 29, 40^ | 2 studies ^20, 47^ |  |
| Mobile app | 1 study ^18^ |  |  |  |  |  |  | 1 study ^8^ | 1 study ^35^ | 2 studies ^28, 44^ |  |
| Tablet App |  |  |  |  |  |  |  |  |  | 1 study ^43^ |  |
| Virtual reality |  |  | 2 studies ^32, 37^ |  |  |  |  |  |  |  |  |
| Computer software | 1 study ^34^ |  |  |  |  |  |  |  |  | 1 study ^15^ |  |
| Website and email |  |  |  |  | 1 study ^30^ | 1 study ^16^ |  |  |  | 1 study ^26^ |  |
| Teleconference and app |  |  |  |  | 1 study ^7^ |  |  |  |  |  |  |
| Teleconference and website | 1 study ^6^ |  |  |  |  |  |  |  |  |  |  |
| Teleconference and social media |  |  |  | 1 study ^31^ | 1 study ^24^ |  |  |  |  |  |  |
| App, website and email |  |  |  |  |  |  |  |  |  |  | 1 study ^46^ |

*HRQoL – Health related quality of life*

*QoL – Quality of Life*

**Supplementary material 7:** List of Abbreviations

BDI – Beck depression inventory

CBT – Cognitive behavioural therapy

CH – Connected health

CT – Clinical trial

EDSS – Expanded disability status scale

HADS – Hospital anxiety and depression scale

HRQoL – Health related quality of life

HRQoL – Health related quality of life

MMAT – Mixed methods appraisal tool

MOOC – Massive open online course

MS – Multiple sclerosis

MSIS-29 – Multiple sclerosis impact scale

PDDS – Patient determined disease scale

PHQ-9 – Patient health questionnaire

PPMS – Primary progressive multiple sclerosis

PRISMA – Preferred reporting items for systematic review and meta-analysis

PROSPERO – International prospective register of systematic reviews

PWMS – People with multiple sclerosis

QoL – Quality of life

QoL – Quality of life

RCT – Randomized control trial

RRMS – Relapsing-remitting multiple sclerosis

SPMS – Secondary progressive multiple sclerosis

VR – Virtual reality

References of Included Articles

1. Alschuler KN, Arewasikporn A, Nelson IK, et al. Promoting Resilience in Individuals Aging With Multiple Sclerosis: Results From a Pilot Randomized Controlled Trial. *REHABILITATION PSYCHOLOGY* 2018; 63: 338-348. DOI: doi:10.1037/rep0000223.

2. Bessing B, van der Mei I, Taylor BV, et al. Evaluating the impact of the Understanding Multiple Sclerosis online course on participant MS knowledge, health literacy, resilience, self-efficacy, quality of life, and MS symptom severity. *MULTIPLE SCLEROSIS AND RELATED DISORDERS* 2022; 60. DOI: doi:10.1016/j.msard.2022.103717.

3. Boeschoten RE, Nieuwenhuis MM, van Oppen P, et al. Feasibility and outcome of a web-based self-help intervention for depressive symptoms in patients with multiple sclerosis: A pilot study. *Journal of the Neurological Sciences* 2012; 315: 104-109. DOI: doi:10.1016/j.jns.2011.11.016.

4. Bogosian A, Chadwick P, Windgassen S, et al. Distress improves after mindfulness training for progressive MS: A pilot randomised trial. *Multiple sclerosis (Houndmills, Basingstoke, England)* 2015; 21: 1184-1194. DOI: doi:10.1177/1352458515576261.

5. Bulbul SB, Keser I, Yucesan C, et al. Effects of pelvic floor muscle training applied with telerehabilitation in patients with multiple sclerosis having lower urinary track symptoms: A randomized controlled trial. *Health care for women international* 2023: 1-17. DOI: doi:10.1080/07399332.2023.2190593.

6. Cavalera C, Rovaris M, Mendozzi L, et al. Online meditation training for people with multiple sclerosis: A randomized controlled trial. *MULTIPLE SCLEROSIS JOURNAL* 2019; 25: 610-617. DOI: doi:10.1177/1352458518761187.

7. Chen MH, Cherian C, Elenjickal K, et al. Real-time associations among MS symptoms and cognitive dysfunction using ecological momentary assessment. *FRONTIERS IN MEDICINE* 2023; 9. DOI: doi:10.3389/fmed.2022.1049686.

8. Chikersal P, Venkatesh S, Masown K, et al. Predicting Multiple Sclerosis Outcomes During the COVID-19 Stay-at-home Period: Observational Study Using Passively Sensed Behaviors and Digital Phenotyping. *JMIR MENTAL HEALTH* 2022; 9. DOI: doi:10.2196/38495.

9. Claflin SB, Campbell J and Taylor BV. Healthcare utilisation and perceived healthcare accessibility and quality amongst people living with multiple sclerosis enroled in an online course. *Mult Scler Relat Disord* 2023; 73: 104621. DOI: doi:10.1016/j.msard.2023.104621.

10. Claflin SB, Mainsbridge C, Campbell J, et al. Self-reported behaviour change among multiple sclerosis community members and interested laypeople following participation in a free online course about multiple sclerosis. *Health promotion journal of Australia : official journal of Australian Association of Health Promotion Professionals* 2022; 33: 768-778. DOI: doi:10.1002/hpja.559.

11. Dogru-Huzmeli E, Duman T, Cakmak AI, et al. Can diplopia complaint be reduced by telerehabilitation in multiple sclerosis patient during the pandemic?: A case report. *Neurological Sciences* 2021; 42: 4387-4390. DOI: doi:10.1007/s10072-021-05194-2.

12. Donkers SJ, Nickel D, Paul L, et al. Adherence to Physiotherapy-Guided Web-Based Exercise for Persons with Moderate-to-Severe Multiple Sclerosis: A Randomized Controlled Pilot Study. *Int J MS Care* 2020; 22: 208-214. DOI: doi:10.7224/1537-2073.2019-048.

13. Dunne J, Chih HJ, Begley A, et al. A randomised controlled trial to test the feasibility of online mindfulness programs for people with multiple sclerosis. *Multiple Sclerosis and Related Disorders* 2021; 48. DOI: doi:10.1016/j.msard.2020.102728.

14. Fischer A, Schroder J, Vettorazzi E, et al. An online programme to reduce depression in patients with multiple sclerosis: a randomised controlled trial. *LANCET PSYCHIATRY* 2015; 2: 217-223. DOI: doi:10.1016/S2215-0366(14)00049-2.

15. Flachenecker P, Bures AK, Gawlik A, et al. Efficacy of an Internet-Based Program to Promote Physical Activity and Exercise after Inpatient Rehabilitation in Persons with Multiple Sclerosis: A Randomized, Single-Blind, Controlled Study. *INTERNATIONAL JOURNAL OF ENVIRONMENTAL RESEARCH AND PUBLIC HEALTH* 2020; 17. DOI: doi:10.3390/ijerph17124544.

16. Gandy M, Heriseanu AI, Balakumar T, et al. The wellbeing neuro course: a randomised controlled trial of an internet-delivered transdiagnostic psychological intervention for adults with neurological disorders. *PSYCHOLOGICAL MEDICINE* 2022. DOI: doi:10.1017/S0033291723000338.

17. Gandy M, Karin E, McDonald S, et al. A feasibility trial of an internet-delivered psychological intervention to manage mental health and functional outcomes in neurological disorders. *Journal of Psychosomatic Research* 2020; 136. DOI: doi:10.1016/j.jpsychores.2020.110173.

18. Golan D, Sagiv S, Glass-Marmor L, et al. Mobile-phone-based e-diary derived patient reported outcomes: Association with clinical disease activity, psychological status and quality of life of patients with multiple sclerosis. *PLoS One* 2021; 16: e0250647. DOI: doi:10.1371/journal.pone.0250647.

19. Halstead EJ, Leavitt VM, Fiore D, et al. A feasibility study of a manualized resilience-based telehealth program for persons with multiple sclerosis and their support partners. *Multiple Sclerosis Journal - Experimental, Translational and Clinical* 2020; 6. DOI: doi:10.1177/2055217320941250.

20. Jeong IC, Karpatkin H and Finkelstein J. Physical Telerehabilitation Improves Quality of Life in Patients with Multiple Sclerosis. *Stud Health Technol Inform* 2021; 284: 384-388. DOI: doi:10.3233/shti210752.

21. Jongen PJ, Sinnige LG, van Geel BM, et al. The interactive web-based program MSmonitor for self-management and multidisciplinary care in multiple sclerosis: concept, content, and pilot results. *PATIENT PREFERENCE AND ADHERENCE* 2015; 9: 1741-1750. DOI: doi:10.2147/PPA.S93783.

22. Kahraman T, Savci S, Ozdogar AT, et al. Physical, cognitive and psychosocial effects of telerehabilitation-based motor imagery training in people with multiple sclerosis: A randomized controlled pilot trial. *Journal of telemedicine and telecare* 2020; 26: 251-260. DOI: doi:10.1177/1357633X18822355.

23. Kever A, Aguerre IM, Vargas W, et al. Feasibility trial of a telehealth support group intervention to reduce anxiety in multiple sclerosis. *Clinical Rehabilitation* 2022; 36: 1305-1313. DOI: doi:10.1177/02692155221107077.

24. Khazaeili M, Hajebi MZ, Mohamadkhani P, et al. The effectiveness of Mindfulness-Based Intervention on anxiety, depression and burden of caregivers of multiple sclerosis patients through web conferencing. *Journal of Practice in Clinical Psychology* 2019; 7: 21-32. DOI: doi:10.32598/jpcp.7.1.21.

25. Kratz AL, Alschuler KN, Williams DA, et al. Development and Pilot Testing of a Web-Based Symptom Management Program for Multiple Sclerosis: My MS Toolkit. *REHABILITATION PSYCHOLOGY* 2021; 66: 224-232. DOI: doi:10.1037/rep0000375.

26. Landtblom AM, Guala D, Martin C, et al. RebiQoL: A randomized trial of telemedicine patient support program for health-related quality of life and adherence in people with MS treated with Rebif. *PLoS One* 2019; 14: e0218453. DOI: doi:10.1371/journal.pone.0218453.

27. Leavitt VM, Riley CS, De Jager PL, et al. eSupport: Feasibility trial of telehealth support group participation to reduce loneliness in multiple sclerosis. *Multiple Sclerosis Journal* 2020; 26: 1797-1800. DOI: doi:10.1177/1352458519884241.

28. Limmroth V, Bayer-Gersmann K, Mueller C, et al. Ascertaining Medication Use and Patient-Reported Outcomes via an App and Exploring Gamification in Patients With Multiple Sclerosis Treated With Interferon beta-1b: Observational Study. *JMIR FORMATIVE RESEARCH* 2022; 6. DOI: doi:10.2196/31972.

29. McArthur AR, Peterson EW, Sosnoff J, et al. Online Delivery of the Individualized Reduction of Falls Intervention for Persons With Multiple Sclerosis Who Use a Wheelchair or Scooter Full-time: A Pilot Study. *Int J MS Care* 2023; 25: 82-90. DOI: doi:10.7224/1537-2073.2022-044.

30. Moss-Morris R, McCrone P, Yardley L, et al. A pilot randomised controlled trial of an Internet-based cognitive behavioural therapy self-management programme (MS Invigor8) for multiple sclerosis fatigue. *BEHAVIOUR RESEARCH AND THERAPY* 2012; 50: 415-421. DOI: doi:10.1016/j.brat.2012.03.001.

31. Najafi P, Hadizadeh M, Cheong JPG, et al. Effects of Tele-Pilates and Tele-Yoga on Biochemicals, Physical, and Psychological Parameters of Females with Multiple Sclerosis. *J Clin Med* 2023; 12. DOI: doi:10.3390/jcm12041585.

32. Pagliari C, Di Tella S, Jonsdottir J, et al. Effects of home-based virtual reality telerehabilitation system in people with multiple sclerosis: A randomized controlled trial. *JOURNAL OF TELEMEDICINE AND TELECARE* 2021. DOI: doi:10.1177/1357633X211054839.

33. Paul L, Coulter EH, Miller L, et al. Web-based physiotherapy for people moderately affected with Multiple Sclerosis; quantitative and qualitative data from a randomized, controlled pilot study. *Clinical Rehabilitation* 2014; 28: 924-935. DOI: doi:10.1177/0269215514527995.

34. Pottgen J, Friede T, Lau S, et al. Managing neuropsychological impairment in multiple sclerosis - Controlled study on a standardized metacognitive intervention (MaTiMS). *MULTIPLE SCLEROSIS AND RELATED DISORDERS* 2022; 59. DOI: doi:10.1016/j.msard.2022.103687.

35. Pratap A, Grant D, Vegesna A, et al. Evaluating the Utility of Smartphone-Based Sensor Assessments in Persons With Multiple Sclerosis in the Real-World Using an App (elevateMS): Observational, Prospective Pilot Digital Health Study. *JMIR Mhealth Uhealth* 2020; 8: e22108. DOI: doi:10.2196/22108.

36. Sadeghi N, Eelen P, Nagels G, et al. Innovating Care in Multiple Sclerosis: Feasibility of Synchronous Internet-Based Teleconsultation for Longitudinal Clinical Monitoring. *Journal of Personalized Medicine* 2022; 12. DOI: doi:10.3390/jpm12030433.

37. Saladino ML, Gualtieri C, Scaffa M, et al. Neuro rehabilitation effectiveness based on virtual reality and tele rehabilitation in people with multiple sclerosis in Argentina: Reavitelem study. *Multiple Sclerosis and Related Disorders* 2023; 70. DOI: doi:10.1016/j.msard.2023.104499.

38. Sangelaji B, Smith C, Paul L, et al. Promoting physical activity engagement for people with multiple sclerosis living in rural settings: A proof-of-concept case study. *European Journal of Physiotherapy* 2017; 19: 17-21. DOI: doi:10.1080/21679169.2017.1381306.

39. Sesel AL, Sharpe L, Beadnall HN, et al. A randomized controlled trial of a web-based mindfulness programme for people with MS with and without a history of recurrent depression. *Multiple Sclerosis Journal* 2022; 28: 1392-1401. DOI: doi:10.1177/13524585211068002.

40. Tarakci E, Tarakci D, Hajebrahimi F, et al. Supervised exercises versus telerehabilitation. Benefits for persons with multiple sclerosis. *Acta Neurologica Scandinavica* 2021; 144: 303-311. DOI: doi:10.1111/ane.13448.

41. Turkowitch D, Ludwig R, Nelson E, et al. Telehealth-Delivered Cognitive Behavioral Therapy for Insomnia in Individuals with Multiple Sclerosis: A Pilot Study. *Multiple Sclerosis International* 2022; 2022. DOI: doi:10.1155/2022/7110582.

42. Turner AP, Hartoonian N, Sloan AP, et al. Improving Fatigue and Depression in Individuals With Multiple Sclerosis Using Telephone-Administered Physical Activity Counseling. *JOURNAL OF CONSULTING AND CLINICAL PSYCHOLOGY* 2016; 84: 297-309. DOI: doi:10.1037/ccp0000086.

43. Van Beek JJW, Lehnick D, Pastore-Wapp M, et al. Tablet app-based dexterity training in multiple sclerosis (TAD-MS): a randomized controlled trial. *DISABILITY AND REHABILITATION-ASSISTIVE TECHNOLOGY* 2022. DOI: doi:10.1080/17483107.2022.2131915.

44. Van Geel F, Geurts E, Abasiyanik Z, et al. Feasibility study of a 10-week community -based program using the WalkWithMe application on physical activity, walking, fatigue and cognition in persons with Multiple Sclerosis. *MULTIPLE SCLEROSIS AND RELATED DISORDERS* 2020; 42. DOI: doi:10.1016/j.msard.2020.102067.

45. Van Kessel K, Wouldes T and Moss-Morris R. A New Zealand pilot randomized controlled trial of a web-based interactive self-management programme (MSInvigor8) with and without email support for the treatment of multiple sclerosis fatigue. *CLINICAL REHABILITATION* 2016; 30: 454-462. DOI: doi:10.1177/0269215515584800.

46. Wingo BC, Rinker JR, Goss AM, et al. Feasibility of improving dietary quality using a telehealth lifestyle intervention for adults with multiple sclerosis. *MULTIPLE SCLEROSIS AND RELATED DISORDERS* 2020; 46. DOI: doi:10.1016/j.msard.2020.102504.

47. Zissman K, Lejbkowicz I and Miller A. Telemedicine for multiple sclerosis patients: Assessment using Health Value Compass. *Multiple Sclerosis Journal* 2012; 18: 472-480. DOI: doi:10.1177/1352458511421918.
